# Harnessing synergistic effects of MMP-2 Inhibition and bFGF to simultaneously preserve and vascularize cardiac extracellular matrix after myocardial infarction

**DOI:** 10.1016/j.actbio.2024.10.050

**Published:** 2024-11-12

**Authors:** Hong Niu, Zhongting Liu, Ya Guan, Jiaxing Wen, Yu Dang, Jianjun Guan

**Affiliations:** a Department of Mechanical Engineering and Materials Science, Washington University in St. Louis, St. Louis, MO 63130, USA; b Center of Regenerative Medicine, School of Medicine, Washington University in St. Louis, St. Louis, MO 63110, USA; c Institute of Materials Science and Engineering, Washington University in St. Louis, St. Louis, MO 63130, USA

**Keywords:** Myocardial infarction, MMP-2 inhibitors, Vascularization, local delivery, ROS-scavenging

## Abstract

Myocardial infarction (MI) leads to cardiac extracellular matrix (ECM) degradation and fibrosis, reducing heart function. Consequently, simultaneously addressing ECM degradation and inhibiting cardiac fibrosis is essential for preserving heart function and mitigating adverse remodeling. However, the preserved ECM becomes unstable if not vascularized, as its structure and composition undergo changes over time. ECM vascularization is crucial to improve cardiac function. Presently, there is no clinically approved therapy that can simultaneously preserve and vascularize the ECM, and inhibit cardiac fibrosis. Our study develops a drug delivery system aiming to achieve these goals. It includes the peptide CTTHWGFTLC (CTT), a specific MMP-2 inhibitor, and basic fibroblast growth factor (bFGF), a potent factor with pro-angiogenic and anti-fibrotic properties. An injectable hydrogel serves as the carrier, featuring a rapid gelation that allows for the substantial retention of drugs. Additionally, the hydrogel has the capability to scavenge upregulated reactive oxygen species (ROS), thereby reducing tissue inflammation. Our findings indicate that CTT and bFGF synergistically enhance endothelial cell migration and tube formation while inhibiting the differentiation of fibroblasts into myofibroblasts. Upon delivery into hearts, the system significantly decreases MMP-2 level, promotes angiogenesis, attenuates cardiac fibrosis, and alleviates inflammation, resulting in a noteworthy cardiac function improvement.

## Statement of significance

1) This work addresses key challenges in cardiac repair after myocardial infarction (MI), including extracellular matrix (ECM) degradation, vascularization, and fibrosis. 2) We combined an MMP-2/9 inhibitor (CTT) with bFGF to prevent ECM degradation, enhance vascularization, and inhibit fibrosis, providing a comprehensive strategy to improve cardiac function. 3) An injectable hydrogel was developed with rapid gelation and mechanical properties similar to heart tissue, ensuring efficient drug retention and reducing tissue stress. 4) The hydrogel enabled controlled, spatiotemporal release of CTT to dynamically reduce MMP-2/9 activity, and gradually released bFGF to promote angiogenesis and inhibit fibrosis.

## 1. Introduction

After myocardial infarction (MI), there is a significant upsurge in the levels of matrix metalloproteinases (MMPs) in the heart, particularly MMP-2 and MMP-9, with MMP-2 expression exceeding that of MMP-9 by over 30 times. [1–4]. The expression of MMP-2 is not only elevated but also exhibits a spatiotemporal pattern in infarcted hearts. Its activity begins to rise as early as one hour post-MI [5], reaching its peak between two to three weeks [6]. This overexpression could persist for 8 weeks [7]. The increase in MMP activity leads to the excessive breakdown of the cardiac extracellular matrix (ECM), which in turn causes the left ventricular wall to become thinner and potentially rupture, exacerbating cardiac dysfunction [8–11]. Clinically, uncontrolled ECM degradation has been linked to increased mortality [12–14]. Consequently, a primary goal in post-MI treatment is to mitigate this ECM degradation to preserve cardiac function. Inhibiting the activity of MMP-2 within the infarcted area is a targeted therapeutic strategy. Pharmacological MMP inhibitors are frequently used for this purpose, but they often lack specificity, targeting a wide range of MMPs, not just MMP-2, and can have undesirable toxic effects [15–19]. The efficacy of systemic delivery of these inhibitors is limited, primarily due to challenges in delivering adequate concentrations directly to the infarcted area and concerns over the cytotoxicity of these molecules [20]. Additionally, the spatiotemporal specificity of MMP-2 upregulation in the infarcted zone is not adequately addressed by current systemic delivery methods [21]. Therefore, developing non-toxic, highly selective drugs and effective delivery methods for inhibiting MMP-2 activities is crucial.

Post-stabilization of the ECM, vascularizing it is essential for effective tissue repair [22]. If the ECM is not vascularized in a timely manner, its structure and composition may deteriorate, leading to adverse remodeling and scar formation [23–26]. Therefore, it is crucial to simultaneously preserve and vascularize the cardiac ECM after MI. Currently, there are no established clinical therapies for preserving and vascularizing the ECM in humans post-MI. Spontaneous vascularization in infarcted hearts is often insufficient, prompting the development of various strategies to enhance vascularization, including the delivery of growth factors [27,28], stem cell transplantation [29,30], and miRNA delivery [31–33]. Direct administration of pro-angiogenic factors such as vascular endothelial growth factor (VEGF) [34,35], basic fibroblast growth factor (bFGF) [36], and platelet-derived growth factor (PDGF) is commonly explored [37,38]. However, the systemic delivery of these factors is inefficient due to their short half-life. Injectable microspheres and hydrogels administered via intramyocardial injection offer controlled and localized delivery, potentially enhancing therapeutic efficacy [39–42]. However, the slow-gelling hydrogels often have relatively low retention, leading to a substantial loss of growth factors during the delivery process [43,44]. Enhancing growth factor retention post-delivery using fast-gelling hydrogels as carriers could potentially increase the efficacy of vascularization.

Cardiac fibrosis, which involves an excessive accumulation of ECM in the infarcted area, is another challenge following MI. It leads to tissue stiffening, adversely affecting heart function. Myofibroblasts, which primarily form through the TGFβ pathway, are central to the development of cardiac fibrosis [45,46]. Current treatments, mainly focusing on the systemic delivery of TGFβ inhibitors or anti-TGFβ antibodies, do not effectively prevent myofibroblast formation [47,48]. TGFβ receptor inhibitors could be a solution, but they are often associated with high toxicity, highlighting the need for less toxic therapeutic options [49]. Utilizing non-toxic and anti-fibrotic growth factors for the therapy may circumvent these toxicity concerns.

In this study, we aim to develop a localized drug delivery system for the infarcted myocardium that preserves and vascularizes ECM, prevents cardiac fibrosis, and enhances cardiac function. We utilized a non-toxic cyclic peptide, CTT (CTTHWGFTLC), which selectively inhibits MMP-2 [50–52]. Additionally, we used bFGF for its pro-angiogenic properties and ability to inhibit TGFβ-induced differentiation of fibroblasts into myofibroblasts [53]. For effective drug retention, we developed a thermosensitive, ROS-scavenging, biodegradable, and biocompatible poly (N-isopropylacrylamide) (PNIPAAm)-based hydrogel. This hydrogel quickly gels at body temperature, targets the infarcted area, and degrades in response to hydrolysis and upregulated ROS in infarcted hearts. Our *in vitro* studies demonstrated that CTT and bFGF work synergistically to promote endothelial cell migration and inhibit myofibroblast formation. In vivo results confirmed that this delivery system effectively preserved and vascularized the ECM, and reduced fibrosis, leading to improved cardiac function.

## 2. Materials and methods

### 2.1. Cell culture and treatment

RCFs were maintained under standard culture conditions at 37 °C and 5 % CO_2_ using Dulbecco’s Modified Eagle’s Medium (DMEM), enriched with 10 % fetal bovine serum (FBS, Atlanta Biologicals) and 1 % penicillin-streptomycin (P/S). Before specific treatments, the fibroblasts underwent serum starvation using a serum-free medium overnight. This was followed by incubation with 10 ng/mL of TGFβ1 for 24 h to induce the fibroblast-to-myofibroblast transition, a critical process in cardiac fibrosis.

For cytotoxicity evaluation, the fibroblasts were seeded onto collagen-coated 96-well plates for 24 h. Subsequently, they were treated with varying concentrations of CTT, ranging from 0 to 100 μg/mL. Cell viability in response to these treatments was determined using the MTT assay. This setup was crucial for establishing the non-toxic range of CTT for subsequent experiments.

In *in vitro* experiments assessing the transition of fibroblasts to myofibroblasts, RCFs were cultured on a two-dimensional collagen gel model. This model involved a mixture of collagen type I (from rat tail, Corning, #354,236), DMEM, and 10 % FBS, solidified by adjusting the pH with 1 M sodium hydroxide solution. The fibroblasts were then exposed to different treatment groups, including bFGF (10 ng/mL) alone, CTT (10 μg/mL) alone, and a combination of both CTT and bFGF. The chosen dosage of CTT was based on the cytotoxicity results and previous research indicating effective concentrations for inhibiting MMP-2 activity in fibroblasts [53]. Similarly, the dosage for bFGF was determined from prior studies [54].

Human umbilical vein endothelial cells (HUVECs) were cultured in EBM^™^−2 endothelial cell basal medium, supplemented with EGM^™^−2 SingleQuots^™^, under standard culture conditions. For cytotoxicity assessments using the MTT assay, HUVECs were seeded in 96-well plates and treated with various concentrations of CTT. This step was crucial to ascertain the safety and tolerance levels of HUVECs to CTT, ensuring the applicability of this peptide in endothelial cell-focused treatments.

### 2.2. Scratch and in vitro angiogenesis assays

The scratch assay, a widely used technique for evaluating cell migration, was conducted following established protocols, as outlined in previous studies [55,56]. HUVECs were cultured in growth medium in 100 mm dishes until they reached approximately 80–90 % confluence. A straight scratch was then created through the cell monolayer using a 200 μL pipette tip, and cellular debris was removed with a medium rinse. The cells were subsequently treated with various experimental conditions using serum-free basal medium. To monitor cell migration, images of the same field were captured at 0, 12, 24, and 48 h using a light microscope. The migration ratio was calculated by measuring the distance between the edges of the scratch at different time points using ImageJ software. These experiments were conducted in triplicate, and a total of eight images were captured for each experimental condition.

For the *in vitro* angiogenesis assay, a 2D collagen gel setup was prepared in a 48-well plate, following protocols established in the literature [55,56]. HUVECs were seeded onto the collagen gel surface at a density of 6 × 10^4^ cells/mL. Various treatment solutions were prepared in EBM and added to the cells: 400 μL EBM with 10 ng/mL TGFβ1, 400 μL of EBM with both 10 ng/mL TGFβ1 and 10 μg/mL CTT, 400 μL of EBM with 10 ng/mL TGFβ1 and 10 ng/mL bFGF, and 400 μL of EBM with 10 ng/mL TGFβ1 combined with both CTT and bFGF. After incubating for 24 h under standard culture conditions, the cells underwent immunofluorescence staining using F-actin and DAPI. The collagen gels were then extracted for imaging using Olympus FV1200 confocal microscopy. The density of the formed lumens was quantified based on six images per experimental group.

### 2.3. Hydrogel synthesis and characterization

#### 2.3.1. Polymer synthesis

The copolymer poly(N-isopropylacrylamide-co-methacrylate poly (ethylene glycol) ester-co-acrylate oligolactide-co-4-(acryloyloxymethyl)-phenylboronic acid pinacol ester) (NIPAAm-co-MAPEG-co-APLA-co-AHPPE) was synthesized through free radical polymerization. Prior to use, the monomer NIPAAm was carefully triple-recrystallized, while MAPEG was prepared for polymerization by removing inhibitors using a column filled with an inhibitor remover. APLA and AHPPE were synthesized following procedures described in the existing literature [55,57,58]. The copolymerization process was carried out under a nitrogen atmosphere at 70 °C for 24 h. The resulting copolymer was initially precipitated in hexane, redissolved in tetrahydrofuran (THF), and further purified by precipitation in diethyl ether, followed by vacuum drying. The yield of this process was approximately 80 %. For the theoretical degradation product polymer PNMAA-D, the constituents NIPAAm, MAPEG, and AAc were used. As a comparative control, a non-ROS-responsive PNMA hydrogel was copolymerized using NIPAAm, MAPEG, and APLA. The chemical structures of these polymers, along with their composition ratios, were verified through ^1^H NMR spectroscopy.

#### 2.3.2. Physical properties of the hydrogels

The synthesized PNMAA polymer was dissolved in Dulbecco’s Phosphate Buffered Saline (DPBS) at a concentration of 6 wt.% with continuous stirring overnight. The hydrogel’s gelling property was evaluated at 37 °C to ensure its suitability for in vivo applications. Its injectability was tested at 4 °C using a 27 G needle, a gauge commonly used for intramuscular injections. The gelation time was assessed by dropping polymer solution onto a glass slide at 37 °C, with continuous monitoring of the transition from a transparent liquid to a fully opaque solid state. The time was recorded as gelation time.

For the degradation test, 200 μL of the hydrogel solution in DPBS was placed into 1.5 mL centrifuge tubes and incubated in a 37 °C water bath, with and without 10 mM H_2_O_2_, for four weeks. The initial weights of the empty tube (w1) and the tube with the hydrogel solution (w2) were recorded. On days 0, 7, 14, 21, and 28, the tubes were taken for lyophilization, and the post-lyophilization dry weight was measured as w3. The percentage of weight remaining was calculated using the formula: (w3−w1)/(w2−w1) × 100 %.

To assess the ROS-scavenging capability of the hydrogel, we conducted Fenton reaction and Pyrogallol assays, which measure the scavenging effects on hydroxyl radicals and superoxide, respectively, as detailed in previous studies [55]. The non-ROS-responsive PNMA hydrogel served as the control in these experiments.

#### 2.3.3. Rheological test

The lower critical solution temperatures (LCSTs) for both PNMAA and PNMAA-D were assessed using an HR-20 rheometer. These experiments spanned a temperature range from 4 °C to 37 °C, during which both the storage modulus (G’) and loss modulus (G”) were evaluated. A 20-mm cone geometry was employed, maintaining a constant strain of 3 % alongside an oscillatory frequency of 1 °C/min. Viscosity measurements were performed at 4 °C, employing a continuous shear rate ramp from 1 to 50 s^−1^.

#### 2.3.4. Cytotoxicity of degradation products

The cytotoxicity of the hydrogel degradation products on RCFs was evaluated using an MTT assay. RCFs were cultured in a 96-well plate and exposed to different concentrations of the degradation product in DMEM, ranging from 0 to 40 mg/mL. After a 24-hour incubation, MTT reagent was added to each well for 4 h. This was followed by the addition of DMSO solvent, and absorbance was measured at 590 nm [59].

#### 2.3.5. Cytocompatibility of the hydrogels

For cytocompatibility testing, PNMAA was dissolved in isopropanol (6 wt.%) and 100 μL of this solution was used to form films in 96-well plates. The plates were then placed in an oven at 60 °C overnight to evaporate the solvent. RCFs were seeded onto these films and treated with 100 μM H_2_O_2_ in complete growth medium. Cell proliferation after 24 h was assessed using the PicoGreen dsDNA assay, following previously described protocols [56,57]. PNMA served as the control hydrogel.

### 2.4. in vitro release kinetics

CTT or bFGF were loaded into the PNMAA hydrogel by adding their respective stock solutions to the hydrogel solution and stirring continuously for 4 h. The 200 μL mixture was then transferred to 1.5 mL centrifuge tubes with low protein binding properties. After equilibrating for 4 h, the supernatant was replaced with 200 μL of release medium (1 % P/S in DPBS). On days 1, 3, 5, 7, 14, 21, and 28, the release medium was collected and replaced with fresh medium. The concentration of CTT released was determined by measuring the fluorescence intensity and compared against a standard curve. bFGF concentrations in the release medium were quantified using a bFGF ELISA kit (Peprotech).

To assess the bioactivity of released CTT in promoting HUVEC migration, the release medium was applied to the cells, and images were captured at 0, 12, and 24 h. Migration ratios were quantified using ImageJ software. The inhibitory effect of released CTT on MMP-2 activity was evaluated using a previously established method [52]. Briefly, rhMMP-2 solution of 60 μL was added to a 96-well plate at a concentration of 12 nM, followed by the addition of 100 μL release medium containing CTT collected at days 1, 7, 14, 21, and 28. After 1 hour of incubation at 37 °C, 30 μL of the MMP-2 substrate III was added at a concentration of 12.5 μM. The mixture was further incubated for 3 h. The fluorescence intensity was measured with excitation at 340 nm and emission at 485 nm, both at 0 and 18 h. For testing the bioactivity of released bFGF in promoting cell survival, RCFs were treated with release medium collected at various time points for 24 h. Untreated RCFs served as the control group for all time points in the treatment groups, while RCFs treated with 1 ng/mL bFGF were used as the reference group for normalization. Cell viability was subsequently assessed using the PicoGreen assay.

### 2.5. MI induction and local drug injection via hydrogels in a murine model

All animal studies were conducted in accordance with the animal care and use guidance established by the National Institutes of Health. The protocol was approved by the Institutional Animal Care and Use Committee (IACUC) at Washington University in St. Louis (approval number 21–0282). For the experiments, female C57BL/6 J mice aged 8 to 10 weeks were procured from the Jackson Laboratory and were systematically assigned to one of the following groups: MI (surgery only), Gel (PNMAA hydrogel), CTT/Gel (CTT at 240 μg/mL encapsulated in hydrogel), bFGF/Gel (bFGF at 15 μg/mL encapsulated in hydrogel), and CTT/bFGF/Gel (combination of CTT and bFGF in hydrogel). Each mouse underwent anesthesia before the surgical procedure, which involved making an incision and opening the chest cavity. The left anterior descending (LAD) artery was located and ligated to induce myocardial infarction. Post-ligation, a sterile hydrogel solution was carefully administered into the affected myocardial region using five distinct injection points. Each injection carried a volume of 20 μL, summing up to a total of 100 μL of the solution for each mouse. Following the hydrogel injections, the surgical openings were sutured, and the mice were subsequently placed on a heating pad to aid in their recovery process. The group that underwent surgery without any therapeutic intervention served as the control for this study. At the day 28 endpoint, the number of surviving animals per group was 7, resulting in an overall survival rate of 81 %. Blood was collected, and hearts were harvested on day 28. A second set of surgeries was performed specifically for collecting blood on day 14, with an overall survival rate of 86 %. Blood was collected from 4 surviving mice in each group.

### 2.6. Echocardiographic analysis

Cardiac function was evaluated using echocardiography. The measurements were made prior to MI and four weeks after MI. The animals were anesthetized using inhalation of 1–3 % isoflurane and positioned in a supine orientation on an electrical heating pad during the procedure. The echocardiographic analysis was carried out using a high-resolution ultrasound imaging system (Vevo-2100, Visualsonics) equipped with a specialized linear transducer. Left ventricular geometry and function were assessed through M-mode measurements using the VevoStrain^™^ Advanced Cardiac software, and ejection fraction and fractional shortening were then calculated.

### 2.7. Blood analysis

Blood samples were carefully drawn from the heart at two time points post-MI: 14 days and 28 days. These samples were then promptly centrifuged at 4 °C at a speed of 300 g for 15 min. The extracted plasma was then analyzed using an ELISA kit specifically for detecting MMP-2 (R&D Systems).

### 2.8. Histological analysis

Four weeks after the surgical procedure, each mouse underwent perfusion with 5 mL of DPBS containing 1 mg/mL heparin. This step was undertaken to ensure maximal dilation of the heart’s vascular structures. Subsequently, the hearts were carefully extracted and fixed in 4 % paraformaldehyde (PFA) at 4 °C overnight for optimal tissue preservation. Following fixation, the infarcted regions of the hearts were meticulously processed for histological examination. This involved embedding the tissues in paraffin and sectioning them into 10 μm thick cross-sections. Staining procedures were then carried out on these sections to visualize various tissue components: Hematoxylin and Eosin (H&E) staining for general tissue architecture, and Picrosirius Red staining for collagen. The stained tissue sections were imaged using an inverted light microscope to capture detailed structural features. Collagen types I and III were identified from PSR images acquired using polarized light microscopy with appropriate band-pass filters [52]. The thickness of the ventricular wall in the infarcted areas was measured and quantified using ImageJ software. This process allowed for the assessment of the extent of cardiac tissue damage and the effectiveness of the therapeutic interventions in mitigating structural changes post-myocardial infarction.

### 2.9. Immunostaining and confocal microscopy

Following the harvesting procedure, heart tissue sections were prepared for immunostaining. Blocking was performed using a solution of 10 % goat serum mixed with 0.3 % Triton X-100 in DPBS. Various primary antibodies were used for specific markers: anti-CD31 (Abcam, ab28364) for endothelial cells, anti-α-SMA (Abcam, ab7817) for smooth muscle cells, anti-PGC1α (Abcam, ab191838) for cell mitochondria activity, anti-myosin heavy chain (MHC, R&D, MAB4470) for cardiomyocytes, anti-laminin (Abcam, ab11575) for basement membrane, anti-CD68 (Abcam, ab234401) for M1 macrophages, and anti-Mannose Receptor (CD206, Abcam, ab64693) for M2 macrophages. The sections were then incubated with corresponding secondary antibodies: Alexa Fluor 488, Alexa Fluor 546, and Alexa Fluor 647. Nuclear staining was performed using DAPI.

For ROS detection, a frozen staining technique was utilized. Heart tissues were perfused with a sucrose solution and subsequently incubated overnight at 4 °C in a mixture containing 70 % PFA and 30 % sucrose. After embedding in the OCT cryostat medium, the tissues were sectioned into 10 μm slices. Staining for ROS detection was conducted using CM-H2DCFDA, along with DAPI for nuclear staining, at room temperature for 1 hour.

The immunostained and ROS-stained heart sections were imaged using an Olympus FV1200 confocal microscope, employing the Z-stack mode to capture detailed structural information. For the quantification of stainings in Figs. 5b, c, h, Figs. 6, and 7a, the number of data points per treatment group was based on 5–8 images captured for the infarcted area. For the quantification of stainings in Fig. 5j, 8–14 images were captured for the remote region instead of the infarcted region. These analyses allowed for the evaluation of various cellular and molecular markers within the infarcted heart tissues, providing valuable insights into the effects of different treatments on cardiac tissue repair and remodeling processes.

### 2.10. Western blotting and ELISA analysis

Protein was isolated from HUVECs following treatment using RIPA lysis buffer supplemented with 1 % Halt^™^ phosphatase inhibitor cocktail (Life Technologies). The lysed cell mixture was then transferred into 1.5 mL centrifuge tubes and gently agitated for 2 h at 4 °C. Centrifugation of the tubes at 12,000 g for 20 min facilitated the collection of the protein, which was subsequently quantified using the Bradford protein assay kit.

For the immunoblotting process, cell or tissue lysates were prepared as per established protocols [55]. Proteins were first separated using 10 % precast gels (Bio-Rad) and then electro-transferred onto immune-blot polyvinylidene difluoride (PVDF) membranes. To prevent non-specific binding, the membranes were blocked and then incubated with a primary antibody overnight at 4 °C. The primary antibodies included phosphorylated Smad1/5/8 (Sigma-Aldrich, AB3848) and MT1-MMP (Cell Signaling, D1E4). GAPDH (Cell Signaling, D16H11) served as a loading control to ensure equal protein loading across samples.

Subsequently, membranes were incubated with horseradish peroxidase (HRP)-conjugated secondary antibodies (Life Technologies). The immunoblots were visualized using the WesternBright HRP substrate detection kit (Advansta) and imaged with the ChemiDoc XPS system. This process enabled the detection and analysis of specific protein expressions, particularly the phosphorylation status of Smad1/5/8, providing insights into the cellular responses to various treatments.

### 2.11. Real-time RT-PCR

For *in vitro* studies, RNA was extracted from cells using TRIzol reagent. For in vivo samples, RNA from infarcted heart tissues was isolated by immersing the tissues in TRIzol. The samples were then homogenized on ice using an ultra-homogenizer. RNA isolation involved a chloroform extraction step, followed by purification with isopropyl alcohol and 70 % ethanol. The purified RNA was dissolved in nuclease-free water. Its concentration and quality were assessed using a Nanodrop spectrophotometer.

The extracted RNA was reverse-transcribed into complementary DNA (cDNA) for RT-PCR analysis using a cDNA synthesis kit (Applied Biosystems), following the manufacturer’s instructions. Primers used for RT-PCR (Sigma-Aldrich) are detailed in Table S1. For the PCR reactions, Maxima SYBR Green/Fluorescein master mix (Life Technologies) was used, along with the appropriate primers and cDNA in a 384-well plate format. PCR was conducted on the QuantStudio Real-Time PCR System (Life Technologies). The reaction mixture contained 0.3 μL cDNA, 2 μL SYBR mix, 5.3 μL nuclease-free water, and 2.4 μL of the primers. The PCR protocol included an initial denaturation step at 95 °C for 3 min, followed by 45 cycles of amplification, which involved denaturation, annealing, extension, and fluorescence data collection. β-actin was used as an internal control for normalization. The relative expression levels of the target genes were calculated using the ΔΔCt method, allowing for comparison of gene expression across different samples.

### 2.12. Statistical analysis

Data were expressed as mean ± standard deviation. Statistical comparisons were done using one-way or two-way ANOVA followed by the Bonferroni post-test, with a significance level set at *p* < 0.05.

## 3. Results and discussion

### 3.1. CTT and bFGF modulated migration of HUVECs, promoted pro-angiogenic growth factors, and reduced the activation of myofibroblasts

**Fig. 1.**
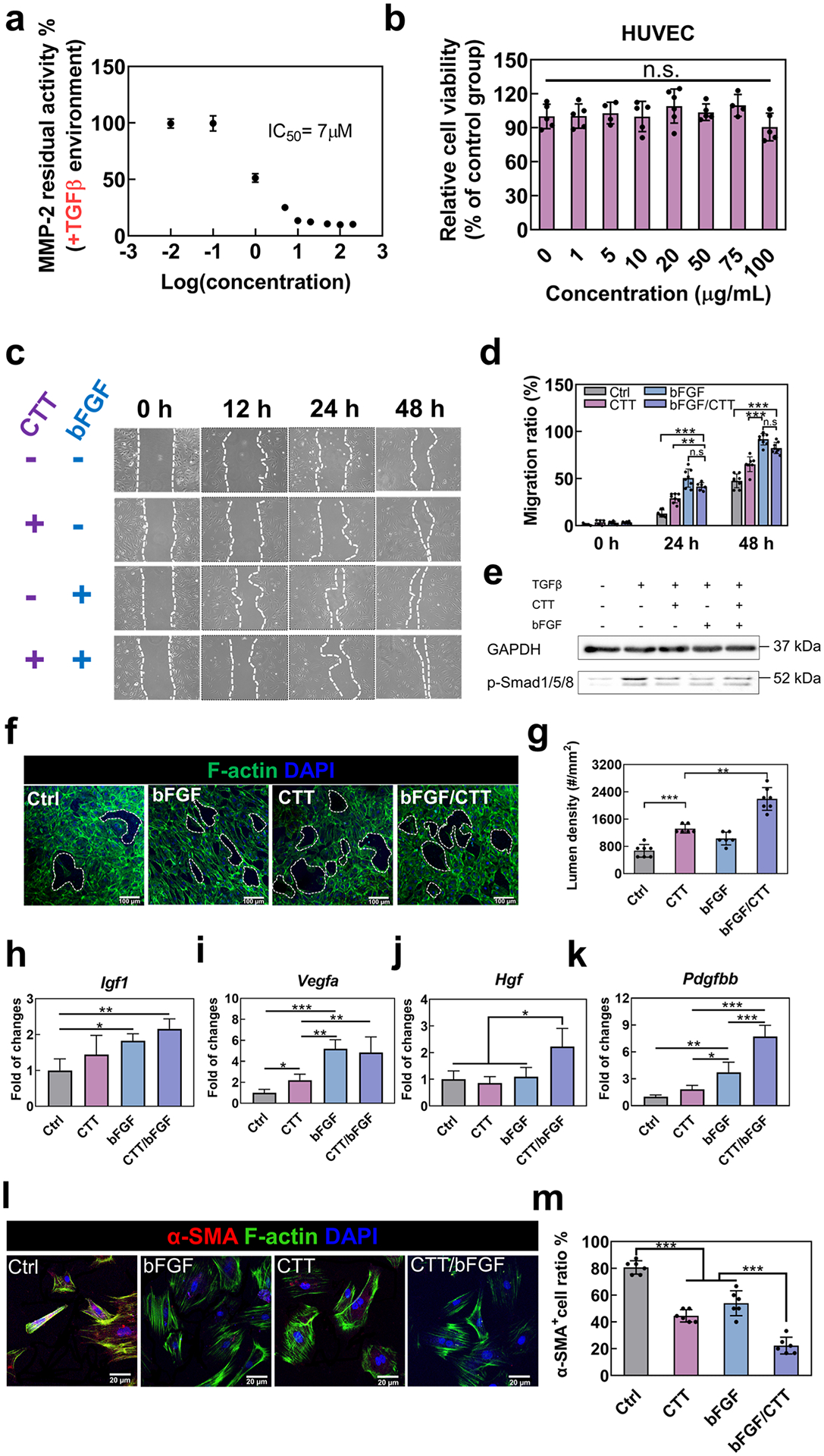
CTT and bFGF modulate the functions of HUVECs and reduce the activation of myofibroblasts. a. Concentration–dependence of CTT in inhibiting MMP-2 activity. b. Cell viability of HUVECs cultured with different concentrations of CTT tested by MTT assay. c. Representative images of HUVEC migration for 48 h in an environment where TGFβ was added (*n* = 8 images per condition). d. Quantification of migration ratio based on the images. e. Immunoblotting of p-Smad1/5/8 and GAPDH expression derived from HUVECs. HUVECs were treated with serum-free medium (control), TGFβ (10 ng/mL), CTT (10 μg/mL), and bFGF (1 μg/mL). f. Tube formation assay using HUVECs cultured on a 2D collagen model. g. Quantification of tube density. h-k. Gene expression of angiogenic factors by the cDNA derived from HUVECs with different treatment groups. (h). *IGF1; (i). VEGFA; (j). HGF and (k). PDGFBB*. l. Representative images of RCFs cultured on collagen gel with different treatments (*n* = 8 images per condition). m. Percentage of α-SMA+ cells in the different groups. ^n.s.^ *p* > 0.5, *p < 0.1, **p < 0.01, ***p < 0.001.

The peptide CTT has been identified as a specific inhibitor for MMP-2 [50–52]. and bFGF is known for its role in promoting angiogenesis [53, 60]. We hypothesized that the combined application of CTT and bFGF could simultaneously mitigate cardiac ECM degradation and facilitate the vascularization of the preserved ECM. To test this, we first evaluated the efficacy of CTT in inhibiting MMP-2 activity within a TGFβ-induced environment. As illustrated in Fig. 1a, CTT exhibited significant inhibition of substrate degradation at a concentration of 7 μM (IC50), highlighting its potent efficacy in suppressing MMP-2 activity. We next evaluated the cytotoxicity of various concentrations of CTT on HUVECs using an MTT assay (Fig. 1b). The results showed no significant difference in cell viability across different CTT concentrations (ranging from 0 to 100 μg/mL), indicating that CTT is non-toxic to HUVECs within this concentration spectrum.

Further, we explored the joint effect of CTT and bFGF on the migration of HUVECs, especially in the context of elevated TGFβ levels, a condition commonly associated with MI. Elevated TGFβ is known to disrupt endothelial cell functions, such as migration and morphogenesis, thereby impeding angiogenesis [61–64]. Our observations revealed that bFGF significantly enhanced HUVEC migration. More interestingly, CTT appeared to further augment this migration process (Fig. 1c and d). Notably, the combination of CTT and bFGF exhibited a significantly higher migration ratio compared to the group treated with bFGF alone (*p* < 0.05 at 24 h, and *p* < 0.001 at 48 h), while showing no significant difference from the CTT alone group (*p* > 0.05).

To uncover the mechanisms behind these effects, we conducted immunoblotting on HUVECs treated under various conditions: without TGFβ, with TGFβ, with CTT/TGFβ, bFGF/TGFβ, and CTT/bFGF/TGFβ. We focused on the Smad1/5/8 pathway, which is known to be directly linked to decreased migration of endothelial cells [65]. The results indicated an induction of Smad1/5/8 phosphorylation in response to TGFβ (Fig. 1e and Figure S4a). However, the presence of CTT, bFGF, or their combination alongside TGFβ markedly reduced the phosphorylation of Smad1/5/8 compared to the TGFβ group. These findings collectively suggest that CTT and bFGF, either individually or in combination, effectively enhance HUVEC migration by inhibiting Smad1/5/8 phosphorylation.

To further identify the function of CTT/bFGF on endothelial cells, we performed an *in vitro* tube formation assay by culturing HUVECs on a 2D collagen model. The results demonstrated that both CTT and bFGF independently contributed to an increased lumen formation of HUVECs. Notably, when combined, CTT and bFGF led to a substantially higher density of lumens compared to all other groups (Fig. 1f and g). These findings not only underscore the individual efficacy of CTT and bFGF in promoting endothelial cell morphogenesis but also emphasize the synergistic effect of their combined application.

Following the observed enhancement in cell migration and tube formation with CTT and bFGF treatment, we further investigated their combined effect on the stimulation of pro-angiogenic factors in endothelial cells (Fig. 1h–k). Treatment with CTT alone notably increased the expression of vascular endothelial growth factor A (*VEGFA*) (*p* < 0.05, Fig. 1i). Conversely, bFGF treatment alone was effective in elevating the levels of insulin-like growth factor 1 (*IGF1*) (*p* < 0.01, Fig. 1h) and platelet-derived growth factor-BB (*PDGFBB*) (*p* < 0.01, Fig. 1k), but did not significantly affect hepatocyte growth factor (*HGF*) expression (Fig. 1j). Notably, the combination of CTT and bFGF resulted in a nearly two-fold increase in *HGF* expression compared to other treatment groups, and also showed a superior elevation in *PDGFBB* levels. These findings suggest a strong synergistic effect of CTT and bFGF in promoting the expression of key pro-angiogenic growth factors. bFGF is also known for its anti-fibrotic properties, particularly in inhibiting the formation of myofibroblasts [53,60]. To further explore whether the combined use of CTT and bFGF impacts this anti-fibrotic action, we studied their effect on the differentiation of cardiac fibroblasts into myofibroblasts in a simplified cardiac fibrosis model induced by TGFβ, a potent inducer of myofibroblast differentiation. Exposure to TGFβ1 led to about 80 % of fibroblasts transitioning into myofibroblasts. However, CTT alone significantly inhibited this transition, resulting in a reduced density of myofibroblasts compared to the group treated only with TGFβ1 (Fig. 1l and m). The combination of CTT and bFGF demonstrated an even more pronounced inhibitory effect, markedly reducing myofibroblast density compared to all other treatments. This synergistic effect in reducing TGFβ1-induced myofibroblast formation suggests a potential interaction between MMP-2 inhibition by CTT and the anti-fibrotic action of bFGF. However, the precise mechanisms behind this synergism require further investigation.

### 3.2. Development of an injectable, thermosensitive, fast gelation, and ROS-scavenging hydrogel for delivery of CTT and bFGF

**Fig. 2.**
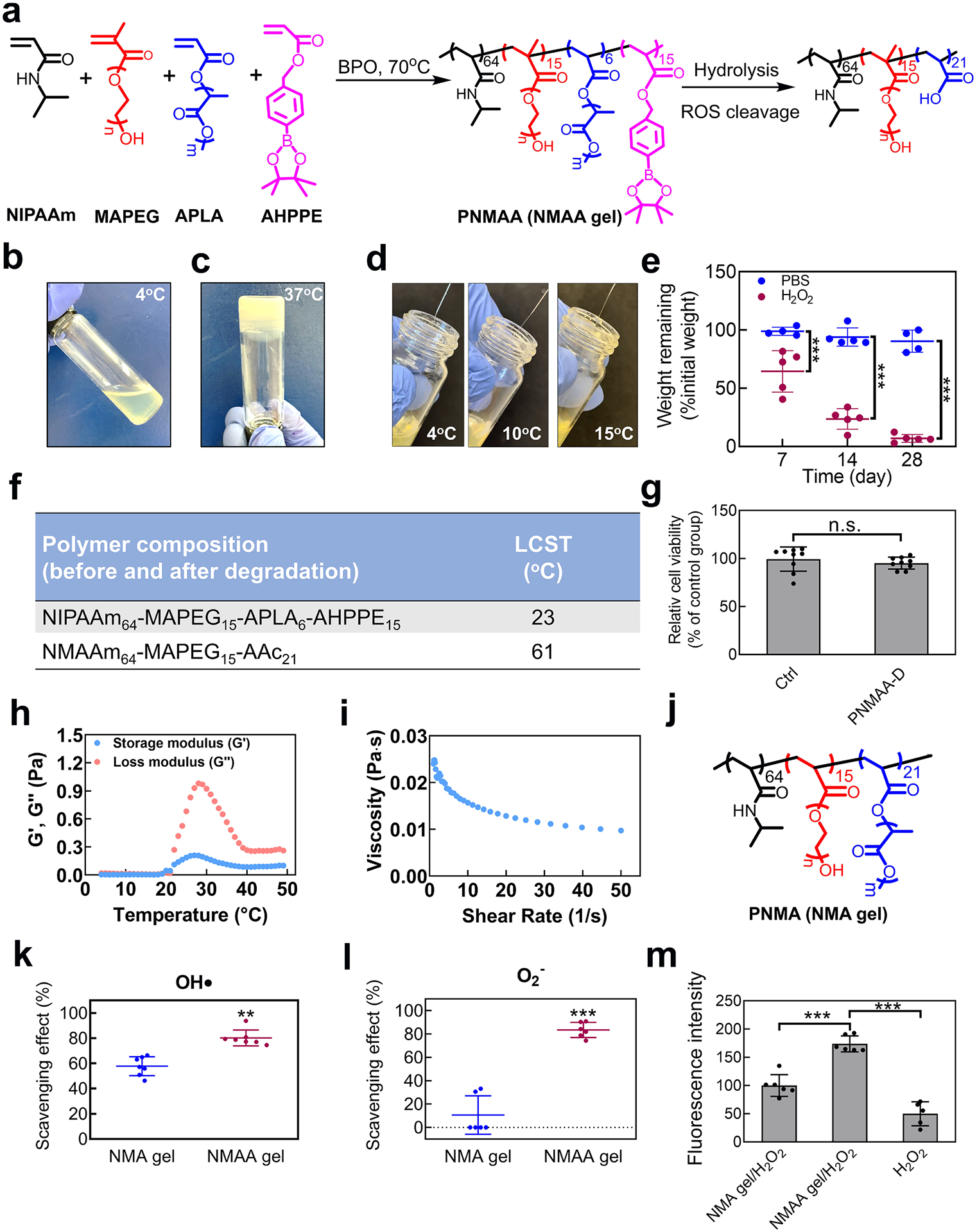
Development of NMAA hydrogel. a. Synthesis of poly (NIPAAm_64_-*co*-MAPEG_15_-*co*-APLA_6_-*co*-AHPPE_15_) by the copolymerization of NIPAAm, MAPEG, APLA, and AHPPE, and hydrolysis of the copolymer. b-d. Physical properties of the NMAA hydrogel, including (b) flowability at 4 °C, (c) gelation at 37 °C, and (d) injectability at 4 °C, 10 °C, and 15 °C. e. Biodegradation of NMAA gel with and without 10 mM H_2_O_2_ for 28 days. f. LCSTs of poly (NIPAAm_65_-*co*-MAPEG_15_-*co*-APLA_5_-*co*-AHPPE_15_) (NMAA) and its theoretical final degradation products poly (NIPAAm_65_-*co*-MAPEG_15_-*co*-AAc_21_) tested by rheometer. g. Cytotoxicity of degradation products collected at day 28 on RCFs. h. The storage modulus (G’) and the loss modulus (G”) of the hydrogel over the temperature range of 4 °C to 49 °C. i. The viscosity of the hydrogel was tested at 4 °C with a shear rate ranging from 1 to 50 *s*^−1^. j. Chemical structure of poly (NIPAAm_64_-*co*-MAPEG_15_-*co*-APLA_21_) that is non-ROS responsive and used as the control gel. k. The scavenging effect on hydroxyl radicals of NMAA hydrogel was determined by the Fenton reaction. Non-ROS responsive APLA gel was used as a control group. l. Scavenging effect on superoxide of NMAA gel tested by Pyrogallol assay. m. RCF proliferation under H_2_O_2_ (100 μM) cultured on NMAA film compared to the non-ROS scavenging film and plate well.

For the targeted delivery of CTT and bFGF into infarcted hearts, we developed a new injectable hydrogel with several key properties: thermosensitivity, rapid gelation, reactive oxygen species (ROS) scavenging, and hydrolytic degradation. The hydrogel was synthesized by copolymerizing NIPAAm, poly (ethylene glycol) methacrylate (MAPEG), 4-(acryloyloxymethyl)-phenylboronic acid pinacol ester (AHPPE), and acrylate-oligolactide (APLA) (Fig. 2a and Figure S1). Each component contributes uniquely: NIPAAm for thermosensitivity, MAPEG for enhanced water solubility, AHPPE for ROS scavenging, and APLA for hydrolysis [56,66,67]. The fast gelation property of the hydrogel is particularly advantageous over those slow gelation hydrogels as it enables rapid solidification upon injection into heart tissue, effectively localizing the encapsulated CTT and bFGF within the tissue. Slow gelation hydrogels can be easily squeezed out after injection [68]. The ROS-scavenging ability is tailored to neutralize elevated ROS levels in infarcted hearts, reducing tissue inflammation. Additionally, the hydrogel was able to undergo hydrolysis, ensuring degradation even in the absence of complete ROS cleavage. After ROS scavenging and hydrolysis, both AHPPE and APLA transform into a highly hydrophilic acrylic acid component, thereby making the final product water soluble (Fig. 2a).

The developed hydrogel (abbreviated as PNMAA) was able to dissolve in DPBS at 4 °C to form a solution (Fig. 2b). The 4 °C solution can quickly solidify (within 22 s) at 37 °C (Fig. 2c). The hydrogel solution was readily injected through 27 G needles (commonly used for muscle injections) at temperatures below its lower critical solution temperature (LCST) of 23 °C, such as at 4 °C, 10 °C, and 15 °C (Fig. 2d). The injectability over a wide temperature range will allow clinicians a time window to apply the hydrogel into infarcted hearts. We also tested the hydrogel degradation under oxidative stress. While there was slight weight loss in DPBS at 37 °C without H_2_O_2_, a significant weight reduction was observed when incubated with 500 μM H_2_O_2_, indicating high ROS responsiveness (***p < 0.001. Fig. 2e). To determine whether the final degradation product can dissolve in body fluid so that it can be removed from the body, we synthesized the theoretical degradation polymer (PNMAA-D), composed of NIPAAm, MAPEG, and acrylic acid (AAc) (Figure S2). This polymer solution had a LCST of 61 °C, suggesting it can be dissolved in the body fluid (Fig. 2f). We further assessed the cytocompatibility of the degradation product by performing a cell viability assay. The results demonstrated that the degradation product was non-toxic (*p* > 0.05. Fig. 2g).

The hydrogel demonstrated transitions of both the storage modulus (G′) and loss modulus (G″) at 22 °C (Fig. 2h). At 37 °C, the values for G′ and G″ were 0.097 Pa and 0.407 Pa, respectively. Furthermore, the hydrogel displayed shear-thinning characteristics, characterized by a reduction in viscosity upon escalating the shear rate from 1 to 50 *s*^−1^ (Fig. 2i).

The ROS scavenging property of the hydrogel was evaluated in terms of its consumption of hydroxyl radicals (HO•) and superoxide (O_2_•^−^) by Fenton assay and Pyrogallol assay, respectively; and a non-ROS scavenging hydrogel, PNMA (NMA gel) was used as the control group (Figs. 2j and S2). The hydrogel scavenged significantly more HO• (*p* < 0.01) and O_2_•^−^ (*p* < 0.001) compared to the control gel lacking the ROS-scavenging AHPPE (Fig. 2k and l). To further evaluate the efficacy of the hydrogel in protecting cells under oxidative stress, the rat cardiac fibroblasts (RCFs) were cultured on hydrogel PNMAA surface, and exposed to 100 μM H_2_O_2_. The cells had a much higher viability compared to those on the control gel surface (*p* < 0.001. Fig. 2m), demonstrating that the ROS scavenging capability protected the cells under oxidative stress.

### 3.3. Sustained release of CTT and bFGF from the hydrogel

**Fig. 3.**
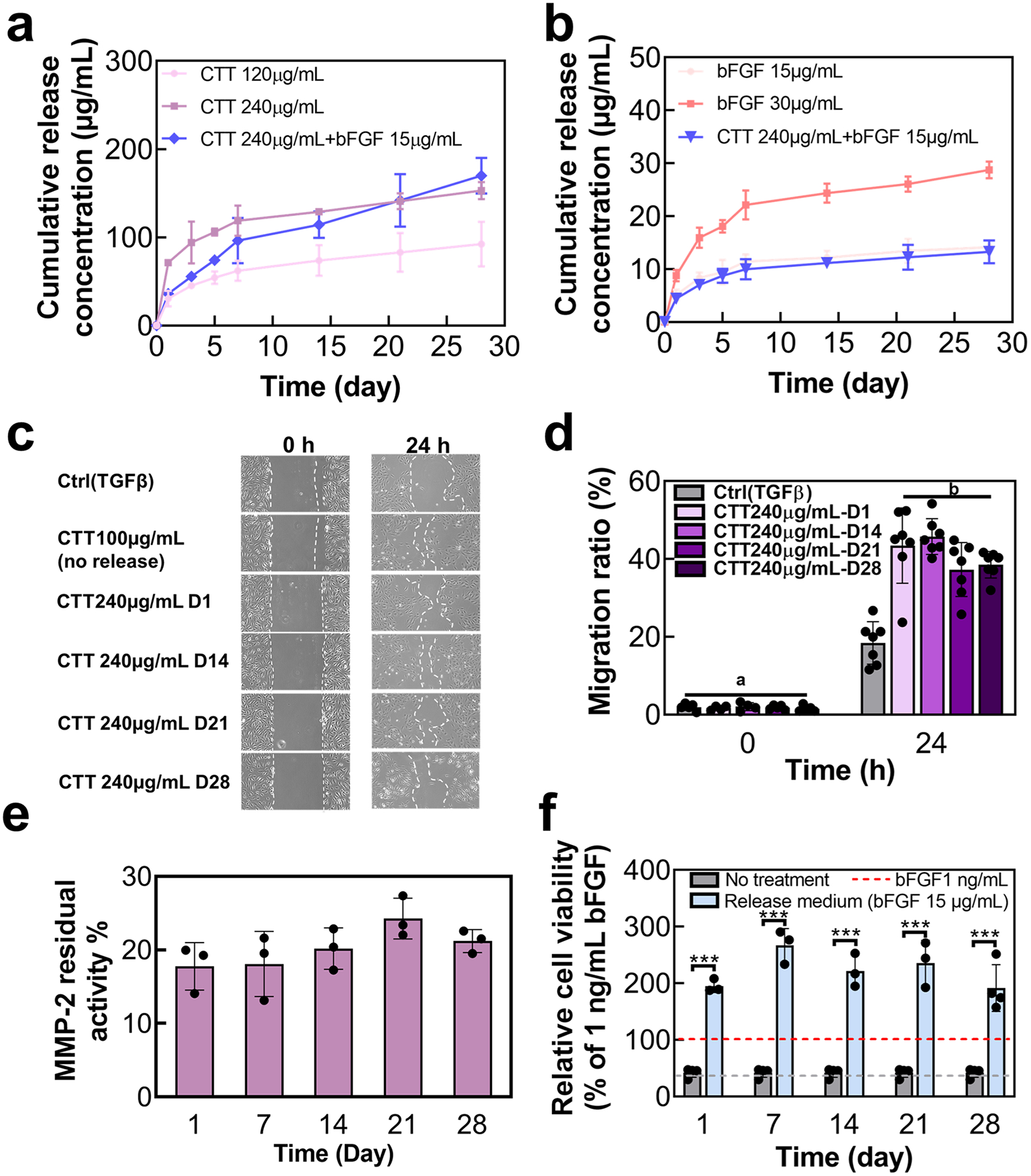
Release of CTT and bFGF encapsulated in NMAA hydrogel for 28 days. a. Release kinetics of CTT at different concentrations and CTT/bFGF in NMAA hydrogel (*n* = 6). b. Release kinetics of bFGF at different concentrations and bFGF/CTT in NMAA hydrogel (*n* = 6). c. Representative images of migration of HUVECs treated with the released medium at days 1, 14, 21, and 28. d. Quantification of migration ratio based on the images. e. Inhibition of MMP-2 by testing residual activity using the released medium collected from days 1, 7, 14, 21, and 28 (*n* = 3). f. Cell proliferation of RCFs treated with the release medium tested by dsDNA assay (*n* ≥ 3).

To encapsulate CTT and bFGF within the PNMAA hydrogel, they were blended into the hydrogel solution at 4 °C. To ensure the stability of bFGF, heparin was added to the mixture to protect it from denaturation. Both CTT and bFGF demonstrated a sustained release from the hydrogel over 28 days (Fig. 3a and b). The release profiles of these agents were dependent on their initial loading amounts within the hydrogel, with higher concentrations leading to greater release. After 4 weeks, hydrogels loaded with 120 μg/mL and 240 μg/mL of CTT released 77.0 % and 63.8 % of their CTT content, respectively. In the case of bFGF, 94.3 % and 95.7 % were released from the hydrogel with 15 μg/mL and 30 μg/mL, respectively. No initial burst release was observed in the release profiles of both bFGF and CTT. This can be attributed to the presence of heparin. Heparin interacts with bFGF and the CTT through electrostatic interactions and hydrogen bonding. Additionally, heparin interacts with the PNIPAAm-based hydrogels via hydrogen bonding [69]. These interactions likely reduced the initial burst release by controlling the dissociation rate of bFGF and CTT from the delivery matrix. Previous studies have shown that incorporating or conjugating heparin into the drug delivery system can decrease or eliminate the initial burst release [70–72]. Interestingly, the release rate of both CTT and bFGF was slightly slower when co-encapsulated in the hydrogel compared to when they were encapsulated separately during the initial three weeks.

To verify the bioactivity of the released CTT and bFGF, we assessed their impact on HUVEC migration under TGFβ conditions. The agents, released on days 1, 14, 21, and 28 (collected from the CTT240 μg/mL group), notably enhanced HUVEC migration (Fig. 3c and d). Additionally, an MMP-2 inhibition assay was performed to confirm whether the released CTT could effectively inhibit MMP-2 activity. The CTT released on the same intervals effectively reduced MMP-2 activity to below 30 % (*p* > 0.05. Fig. 3e). Moreover, the ability of released bFGF to promote RCF proliferation was evaluated. The cell viability was significantly increased when treated with the release medium (collected from the bFGF 15μg/mL group) compared to a control with 1 ng/mL bFGF (*p* < 0.001. Fig. 3f). Collectively, these results indicated that the released CTT and bFGF from PNMAA hydrogel for 28 days were bioactive.

### 3.4. Delivered CTT/bFGF reduced expression of MMP-2 in plasma and tissues, and preserved cardiac ECM

**Fig. 4.**
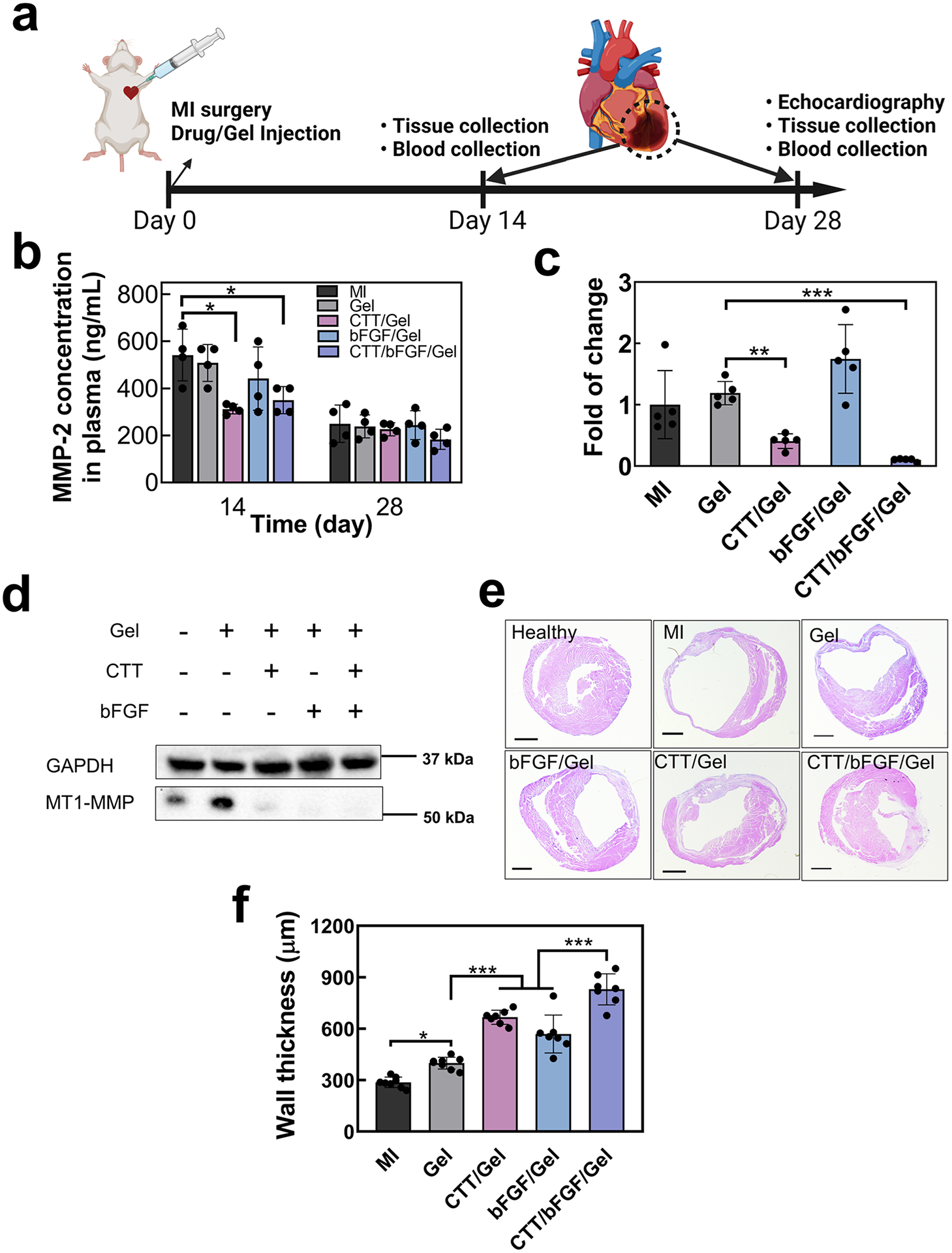
Delivery of CTT/bFGF reduced expression of MMPs 2 and 9 in plasma and tissues and effectively preserved ECM. a. Timeline of the mouse surgery (created with Biorender.com). b. Dynamic change of MMP2 in plasma at days 14 and 28 (*n* = 4). c. In vivo *Mmp2* change in infarcted tissues tested by gene expression (*n* = 5). d. Immunoblotting of α-actinin and MT1-MMP from in vivo tissues. GAPDH was used as a loading control. e. Representative H&E staining images at day 28. f. Quantification of wall thickness based on H&E images (*n* = 7).

To assess the effectiveness of the CTT/bFGF delivery system in treating MI, we utilized a mouse model of acute MI (Fig. 4a). Various treatment groups were injected into the infarcted region, including hydrogel alone (Gel), hydrogel mixed with 240 μg/mL CTT (CTT/Gel), hydrogel mixed with 15 μg/mL bFGF (bFGF/Gel), and a combination of CTT and bFGF in the hydrogel (CTT/bFGF/Gel). The surgery group without any treatment (MI) was used as the control. The injected Gel, CTT/Gel, bFGF/Gel, and CTT/bFGF/Gel quickly solidified in the heart tissue without leaking. To assess the efficacy of released CTT in reducing MMP-2 levels in the heart, we first measured its concentration in plasma, as MMPs from the heart post-MI can enter the bloodstream [73]. On days 14 and 28 post-treatment, blood samples were collected and analyzed for MMP-2 concentrations using ELISA. The results showed no significant difference in MMP-2 levels between the Gel and bFGF/Gel groups compared to the MI group at either time point. However, the CTT/Gel and CTT/bFGF/Gel groups exhibited a significant reduction in MMP-2 concentration at day 14 (*p* < 0.05. Fig. 4b). By day 28, MMP-2 levels decreased across all groups, but no significant differences were observed.

Parallel to the blood analysis, gene expression levels of *Mmp2* in the infarcted hearts were evaluated at day 14, revealing a notable reduction in the CTT/Gel and CTT/bFGF/Gel groups (Fig. 4c). Additionally, the infarcted tissues were analyzed for MMP activity at day 14 using immunoblotting to detect MT1-MMP, an activator of MMPs (Fig. 4d and Figure S4b). MT1-MMP levels were markedly higher in the MI and Gel groups compared to the CTT/Gel, CTT/bFGF/Gel, and bFGF/Gel groups. These findings indicate that the released CTT significantly lowered MMP-2 activity in the infarcted hearts.

The reduction in MMP-2 activity was associated with the preservation of cardiac ECM. Histological examination using H&E staining revealed that the CTT/Gel treatment promoted an increase in left ventricular (LV) wall thickness compared to both the Gel and MI groups (Fig. 4e and f). Notably, the combined treatment with CTT and bFGF further enhanced wall thickness, likely due to CTT preserving ECM and bFGF vascularizing the ECM. Interestingly, the injection of the hydrogel alone (Gel group) also significantly improved wall thickness compared to the MI group, possibly because the hydrogel served as a bulking agent in the infarcted region [74,75]. Moreover, the infarct size quantified from H&E images showed a significant reduction in groups treated with bFGF/Gel and CTT/bFGF/Gel compared to the Gel only group (Figure S5).

### 3.5. Delivered CTT/bFGF enhanced vascularization, and improved cardiac cell survival and proliferation in infarcted area

**Fig. 5.**
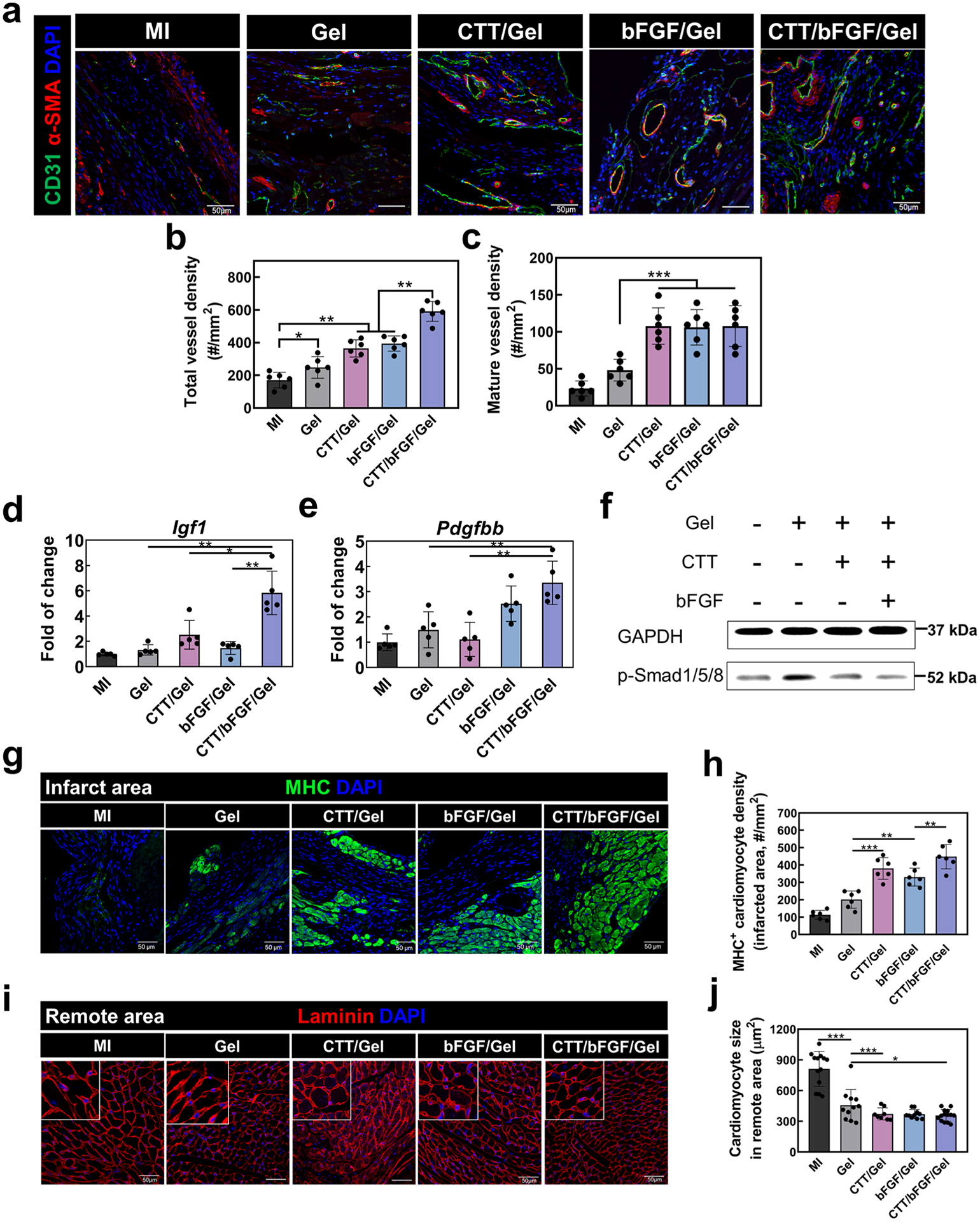
Delivery of CTT/bFGF via NMAA hydrogel effectively improved in vivo vessel formation without causing cardiac fibrosis. a. α-SMA/CD31/DAPI staining for injured heart tissues. Scale bar = 50 μm. b. Quantification of total blood vessel density (α-SMA^−^/CD31^+^ vessels) (*n* = 6). c. Quantification of mature blood vessel (α-SMA^+^/CD31^+^ vessels) density (*n* = 6). d, e. Gene expression of in vivo angiogenic factors including (e) *Igf1*, and (f) *Pdgfbb*. f. Immunoblotting of p-Smad1/5/8 from in vivo tissues. GAPDH was used as a loading control. g. Representative images of MHC/DAPI staining in the infarcted area. Scale bar = 50 μm. h. Quantification of MHC^+^ cell density from IHC images (*n* = 6). i. Representative images of laminin/DAPI staining in remote areas. Scale bar = 50 μm. j. Quantification of remote cardiomyocytes’ size based on laminin signals in the images (*n* = 8–14 based on region size).

To evaluate the efficacy of the CTT/bFGF delivery system in promoting vascularization, we analyzed infarcted heart tissue at day 28 post-MI (Fig. 5a). Both the CTT/Gel and bFGF/Gel treatments significantly increased total vessel density in comparison to the MI and Gel groups (*p* < 0.01), with the bFGF/Gel group showing a slightly higher vessel density than the CTT/Gel group (Fig. 5b). Notably, the CTT/bFGF/Gel group demonstrated the most pronounced increase, suggesting a synergistic effect of the combined CTT and bFGF treatment. This group exhibited approximately three times greater total vessel density relative to the MI group (Fig. 5b). Furthermore, treatments with CTT/Gel, bFGF/Gel, or CTT/bFGF/Gel significantly increased mature vessel density compared to the MI and Gel groups (*p* < 0.001), although no significant differences were observed among these three treatment groups (Fig. 5a and c). In addition to vessel density, we examined the gene expression levels of angiogenic factors in the infarcted areas using real-time RT-PCR (Fig. 5d and e). In line with the vessel density results, the CTT/bFGF combination treatment significantly elevated the expression of *Igf1* and *Pdgfbb*, compared to the other groups. These factors are known for their potent angiogenic properties and have been widely studied in the context of acute MI [76–78].

To understand the enhanced angiogenic effects observed with the combined use of CTT and bFGF, we investigated the phosphorylation levels of Smad1/5/8. This signaling pathway is inversely associated with endothelial cell migration. The simultaneous delivery of CTT and bFGF resulted in lower phosphorylation levels of Smad1/5/8 compared to the treatment with CTT in Gel alone (Fig. 5f and Figure S4c). This finding demonstrates that the improved vascularization achieved through the joint application of CTT and bFGF may stem from an enhanced migration of endothelial cells, which is facilitated by the inhibition of the Smad1/5/8 pathway.

The increased vascularization within the infarcted region had a notable impact on cardiac cell survival. Specifically, the cardiomyocyte density in the groups treated with CTT/Gel, bFGF/Gel, and CTT/bFGF/Gel was significantly higher compared to the MI (*p* < 0.01) and Gel (*p* < 0.001) groups (Fig. 5g and h). In addition, the CTT/bFGF/Gel group showed a slightly higher density of cardiomyocytes than either the CTT/Gel or bFGF/Gel groups. The CTT/bFGF/Gel treatment also significantly increased the density of cells positive for peroxisome proliferator-activated receptor gamma coactivator 1-alpha (PGC-1α), leading to an elevation in mitochondrial biogenesis (Figure S3). These findings align with the observed improvements in vessel density, suggesting that the enhanced vascularization facilitated by the treatments provides an increased supply of nutrients and oxygen, crucial for the survival of cardiomyocytes in the post-infarction environment. Additionally, we investigated the impact of the CTT/bFGF treatment on cardiomyocyte hypertrophy in remote areas of the infarcted heart (Fig. 5i and j). The cardiomyocyte size in the Gel, CTT/Gel, bFGF/Gel, and CTT/bFGF/Gel groups was significantly smaller compared to the MI group. This finding suggests that the treatment effectively reduced cardiomyocyte hypertrophy, which is a common response to myocardial injury.

### 3.6. The delivery system alleviated ROS level in the infarcted area, and delivered CTT/bFGF synergistically modulate in vivo macrophage polarization

**Fig. 6.**
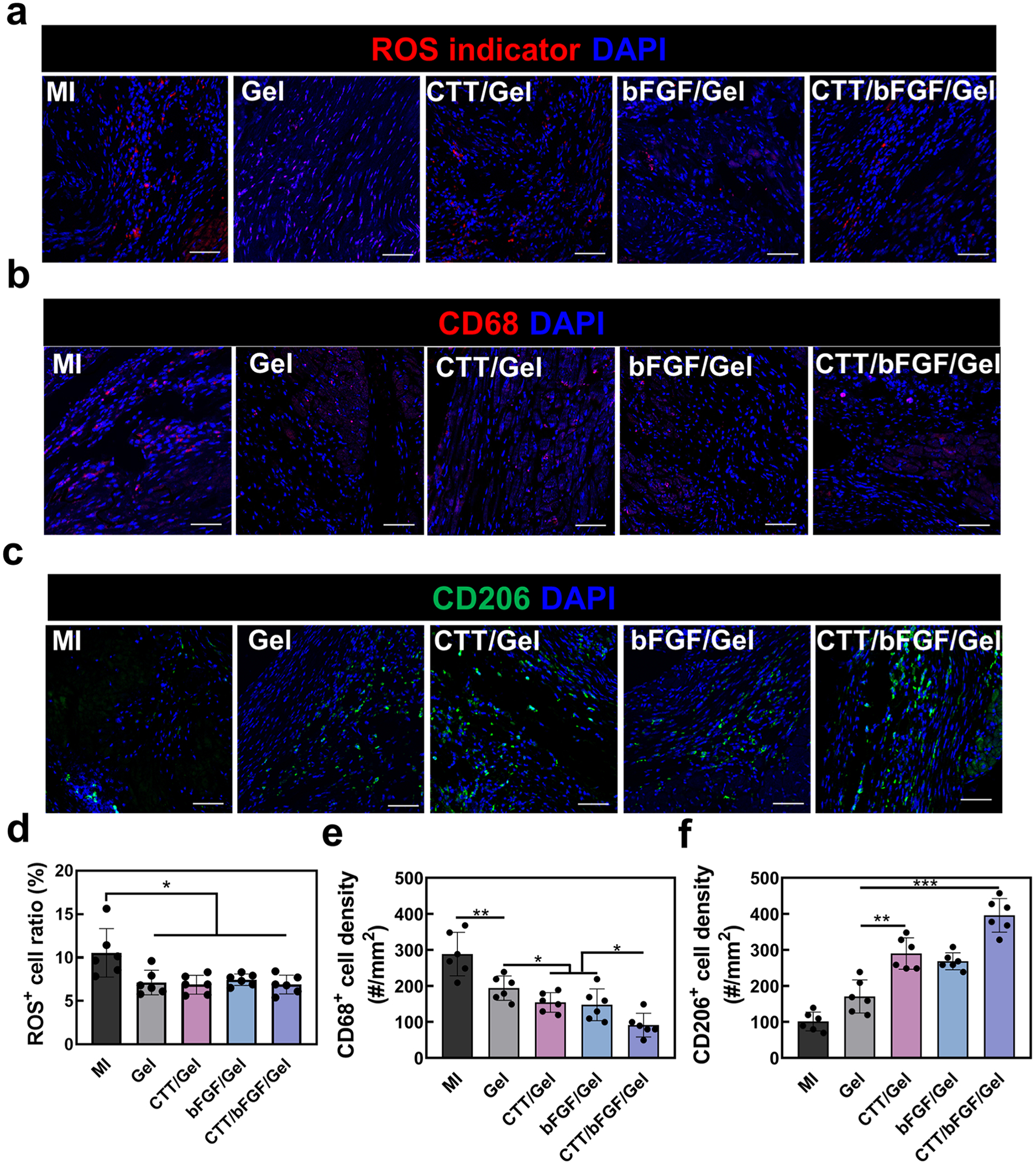
Local inflammation response after the treatment of CTT/bFGF/hydrogel. a. Representative images for CM-H2DCFDA staining. b. Representative images for CD68/DAPI staining. c. Representative images for CD206/DAPI staining. d. Quantification of CM-H2DCFDA^+^ cell density (*n* = 6). e. Quantification of CD68^+^ cell density. f. Quantification of CD206^+^ cell density (*n* = 6). Scale bar = 50 μm.

In hearts affected by MI, elevated levels of ROS can trigger inflammatory responses. To investigate whether our ROS-scavenging hydrogel can reduce ROS levels in the infarcted heart, we performed ROS staining four weeks post-MI. At 4 weeks post-surgery, ROS levels in the infarcted hearts were significantly higher compared to the healthy myocardium of mice without surgery (Figure S6). The groups containing the hydrogel (Gel, CTT/Gel, bFGF/Gel, and CTT/bFGF/Gel) demonstrated a marked decrease in the ratio of ROS+ cells compared to the MI group (*p* < 0.05. Fig. 6a and d), indicating that the ROS-scavenging hydrogel effectively mitigated oxidative stress in the infarcted hearts.

Additionally, we examined the impact of the hydrogel, CTT, and bFGF on macrophage populations in the infarcted hearts by characterizing CD68+ M1 macrophages and CD206+ M2 macrophages. We found that the density of CD68+ inflammatory M1 macrophages was significantly reduced by the hydrogel treatment and further decreased when combined with CTT and bFGF (Fig. 6b and e). Conversely, the density of CD206+ anti-inflammatory M2 macrophages was substantially increased in the groups treated with CTT, bFGF, or their combination (Fig. 6c and f). These findings suggest that the hydrogel not only reduced ROS formation but also that the combination of CTT and bFGF modulated macrophage polarization, favoring a higher density of M2 macrophages and a reduced density of M1 macrophages post-MI. This modulation likely contributes to a more favorable healing environment in the infarcted myocardium.

### 3.7. The delivered CTT/bFGF attenuated cardiac fibrosis, alleviated hypertrophy, and improved cardiac function

**Fig. 7.**
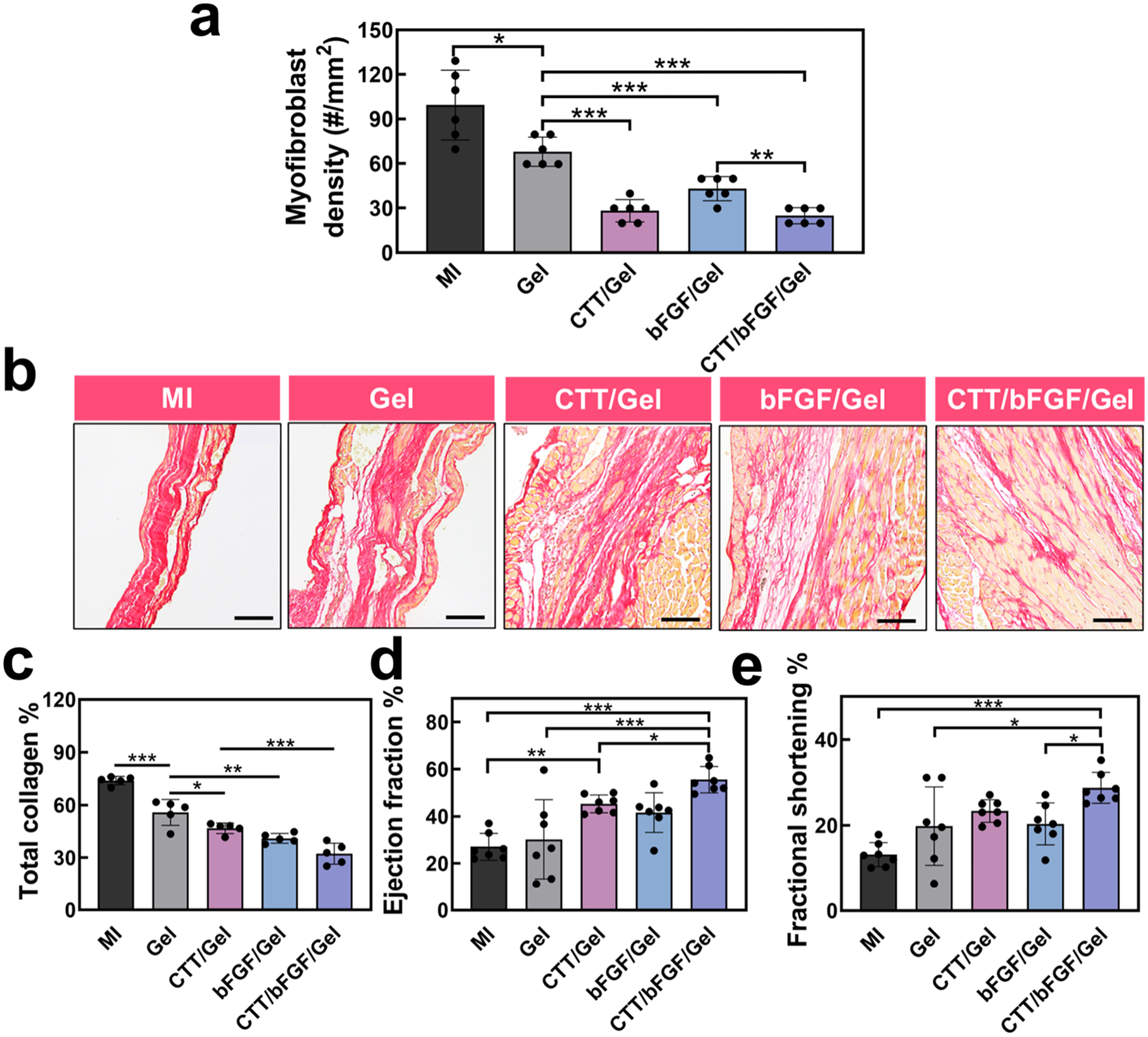
Cardiac regeneration and function repair by the treatment of CTT/bFGF/Gel. a. Myofibroblast density at the injured area based on the images in Fig. 5a (*n* = 6). b. Representative images for tissue samples stained with picrosirius red (PSR). Scale bar = 50 μm. c. Quantification of total collagen deposition calculated from PSR staining images (*n* = 5). d. Echocardiogram analysis of LV ejection fraction 28 days after surgery. e. Echocardiogram analysis of LV fractional shortening (*n* = 7).

To evaluate the potential of the CTT/bFGF delivery system in mitigating cardiac fibrosis, we analyzed the density of myofibroblasts in the infarcted area. Myofibroblasts were identified as ɑSMA+ cells that did not co-localize with CD31+ cells. The application of CTT, bFGF, or their combination significantly reduced myofibroblast density in comparison to the MI and Gel groups (Fig. 7a). These results are consistent with *in vitro* results where CTT and bFGF inhibited cardiac fibroblasts from differentiating into myofibroblasts in the TGFβ condition (Fig. 1l and m). We further assessed collagen deposition in the infarct region using picrosirius red (PSR) staining (Fig. 7b and c). Interestingly, even the Gel group displayed a reduction in total collagen content compared to the MI group. This reduction correlates with the observed decrease in myofibroblast density in the Gel group, possibly indicating that the hydrogel alone, due to its appropriate stiffness, contributed to a decrease in myofibroblast differentiation [79,80]. The use of CTT, bFGF, or their combination with the hydrogel further decreased the total collagen content. Polarized PSR images were used to further characterize collagen types I and III in the infarcted area (Figure S7), both of which are key components of the cardiac ECM. Injection of hydrogel (Gel group) did not significantly affect this ratio. However, injections of CTT/Gel and CTT/bFGF/Gel groups significantly increased the ratio, with an even greater increase observed in the CTT/bFGF/Gel group. A higher proportion of collagen type III may enhance tissue compliance in the infarcted area. The reductions in myofibroblast density, total collagen content, and collagen III/I ratio indicate that the delivery of CTT and bFGF effectively attenuated cardiac fibrosis.

The preservation and vascularization of cardiac ECM, decrease of tissue inflammation, and attenuation of cardiac fibrosis led to a significant improvement in cardiac function. Four weeks post-MI, the groups treated with CTT/Gel or bFGF/Gel exhibited significant enhancements in ejection fraction (EF) and fractional shortening (FS) compared to the MI group (Fig. 7d and e). The combined treatment with CTT and bFGF (CTT/bFGF/Gel group) further amplified these improvements in EF and FS, demonstrating the synergistic benefits of using CTT and bFGF together in post-MI cardiac recovery. Notably, the cardiac function in the CTT/bFGF/Gel group was significantly lower compared to the mice without MI, where EF and FS were 69.9 ± 3.0 % and 38.5 ± 2.4 %, respectively.

The above studies demonstrate that the use of an injectable hydrogel for the co-delivery of CTT and bFGF in treating MI presented several advantages: 1) Cardiac ECM preservation by CTT: CTT played a crucial role in preserving the ECM by inhibiting MMP-2, an enzyme that degrades ECM post-MI. The controlled release of CTT from the hydrogel allowed for a spatiotemporal decrease in MMP-2 activity, aligning with the enzyme’s expression pattern post-MI. MMP-2 activity is known to escalate as early as one hour after MI and peak between two to three weeks post-MI [2,81]. Employing CTT as a specific inhibitor of MMP-2 offers a significant benefit through a targeted mechanism. Unlike broad-spectrum MMP inhibitors, which can affect various members of the MMP family and potentially cause unintended side effects due to their lack of precision [82,83] the specificity of CTT may diminish such risks. This focused approach is likely to enhance the effectiveness of preserving the cardiac ECM by minimizing potential adverse effects. 2) Angiogenesis promotion by bFGF: bFGF was instrumental in fostering angiogenesis, vital for restoring blood flow and facilitating tissue repair. The synergy between CTT and bFGF not only enhanced endothelial cell migration (HUVEC) but also stimulated tube formation. Moreover, the combination upregulated critical angiogenic factors like IGF1 and PDGFBB, amplifying the angiogenic response in the infarcted myocardium. 3) The combined use of CTT and bFGF addresses both ECM preservation and vascularization simultaneously. Most prior studies focus on single-agent therapies, either targeting MMP inhibition or promoting angiogenesis [84–86]. This integrated approach has the potential to significantly improve therapeutic outcomes, presenting a more effective alternative to the limitations of single-agent treatments. 4) Reduction of cardiac fibrosis: Both CTT and bFGF contributed to the reduction of cardiac fibrosis by impeding the transformation of fibroblasts into myofibroblasts. These combined effects culminated in improved cardiac function. Overall, our approach to using a specific MMP inhibitor combined with an angiogenic factor, delivered through an injectable, temperature-sensitive, and ROS-scavenging hydrogel, represents a significant advancement over traditional methods. It addresses multiple facets of MI repair, including ECM preservation, angiogenesis, and anti-fibrosis, potentially offering a more comprehensive and effective treatment strategy.

Despite the above benefits, our studies have limitations in the context of MI treatment: 1) Understanding synergistic mechanisms: Although the synergistic effects of CTT and bFGF are evident, the precise molecular interactions between these two agents, especially in a complex in vivo environment, remain to be fully elucidated. Future studies are planned to delve deeper into these mechanisms. 2) Optimization of dosage and timing: The success of this therapeutic approach is likely contingent on the accurate dosing and timing of the hydrogel delivery. Future research will focus on how variations in dosage and delivery timing influence the reduction in MMP activity, vascularization of the infarcted heart, and attenuation of cardiac fibrosis. 3) Clinical relevance of delivery method: The current model involved direct myocardial injection of the CTT/bFGF delivery system post-acute MI. However, this method poses clinical applicability challenges, including concerns about the feasibility of timely treatment administration and risks associated with myocardial injection in patients with weakened hearts [87,88]. Future studies aim to develop an intravenous (IV) delivery system for CTT/bFGF that can be employed at the acute stage of MI, enhancing the clinical relevance and applicability of this therapeutic strategy.

## 4. Conclusion

In conclusion, we developed a new drug delivery system to simultaneously achieve three goals after MI: preserving cardiac ECM, promoting vascularization, and inhibiting cardiac fibrosis. The system comprised a non-toxic MMP2 specific inhibitor, CTT, and the angiogenic and anti-fibrotic bFGF, along with an injectable, fast gelation, and ROS-scavenging hydrogel. This multifunctional drug delivery system significantly increased cardiac function when applied at the acute MI stage.

## Supplementary Material

Supplemental material
