# Supplementary material for: Harnessing synergistic effects of MMP-2 Inhibition and bFGF to simultaneously preserve and vascularize cardiac extracellular matrix after myocardial infarction
Source: Acta Biomater. Author manuscript; Available in PMC 2025 Jan 1. (PMC11659021; doi:10.1016/j.actbio.2024.10.050)
Supplement: Supplemental material [file NIHMS2041742-supplement-Supplemental_material.docx]

Supplementary Materials

Harnessing synergistic effects of MMP-2 Inhibition and bFGF to simultaneously preserve and vascularize cardiac extracellular matrix after myocardial infarction

Hong Niu, Zhongting Liu, Ya Guan, Yu Dang, Jianjun Guan*

Detailed descriptions of the supporting information figures and tables are included in the main text.


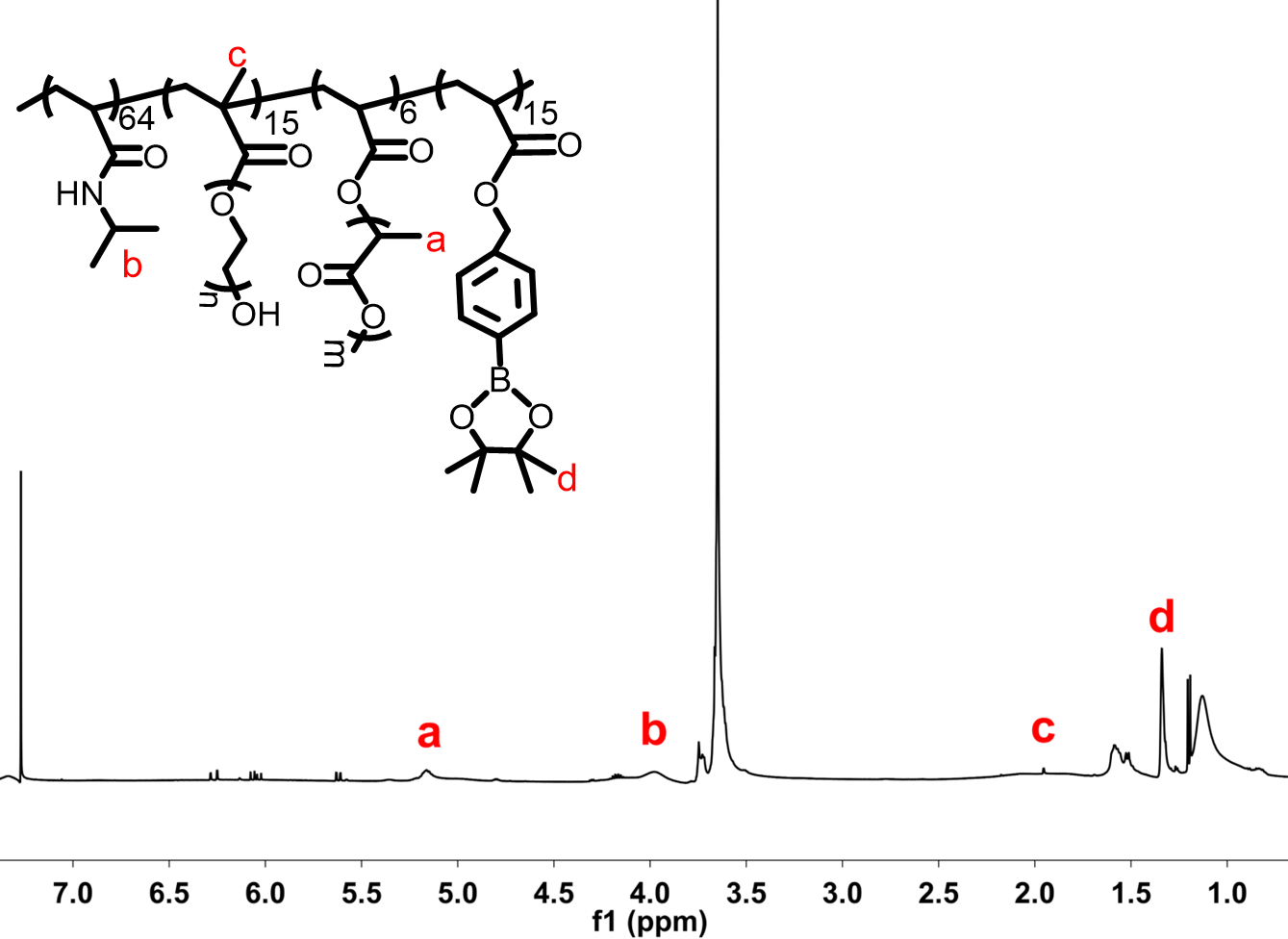


**Figure S1.** NMR of synthesized hydrogel poly (NIPAAm_64_-*co*-MAPEG_15_-*co*-APLA_6_-*co*-AHPPE_15_).


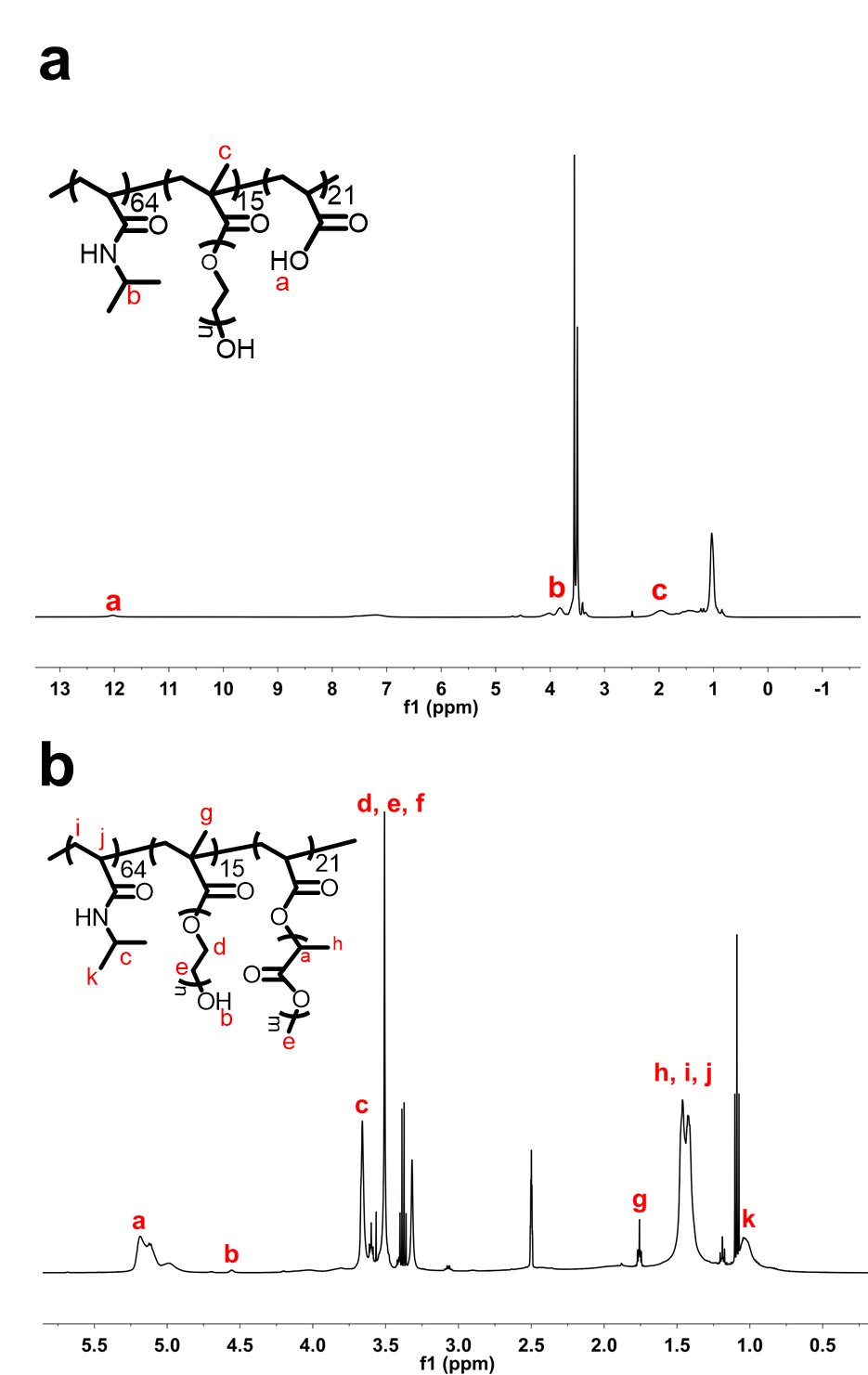


**Figure S2.** NMR of **(a)** synthesized degradation product poly (NIPAAm_64_-*co*-MAPEG_15_-*co*-AAc_21_), and **(b)** synthesized control hydrogel PNMA poly (NIPAAm_64_-*co*-MAPEG_15_-*co*-APLA_21_).


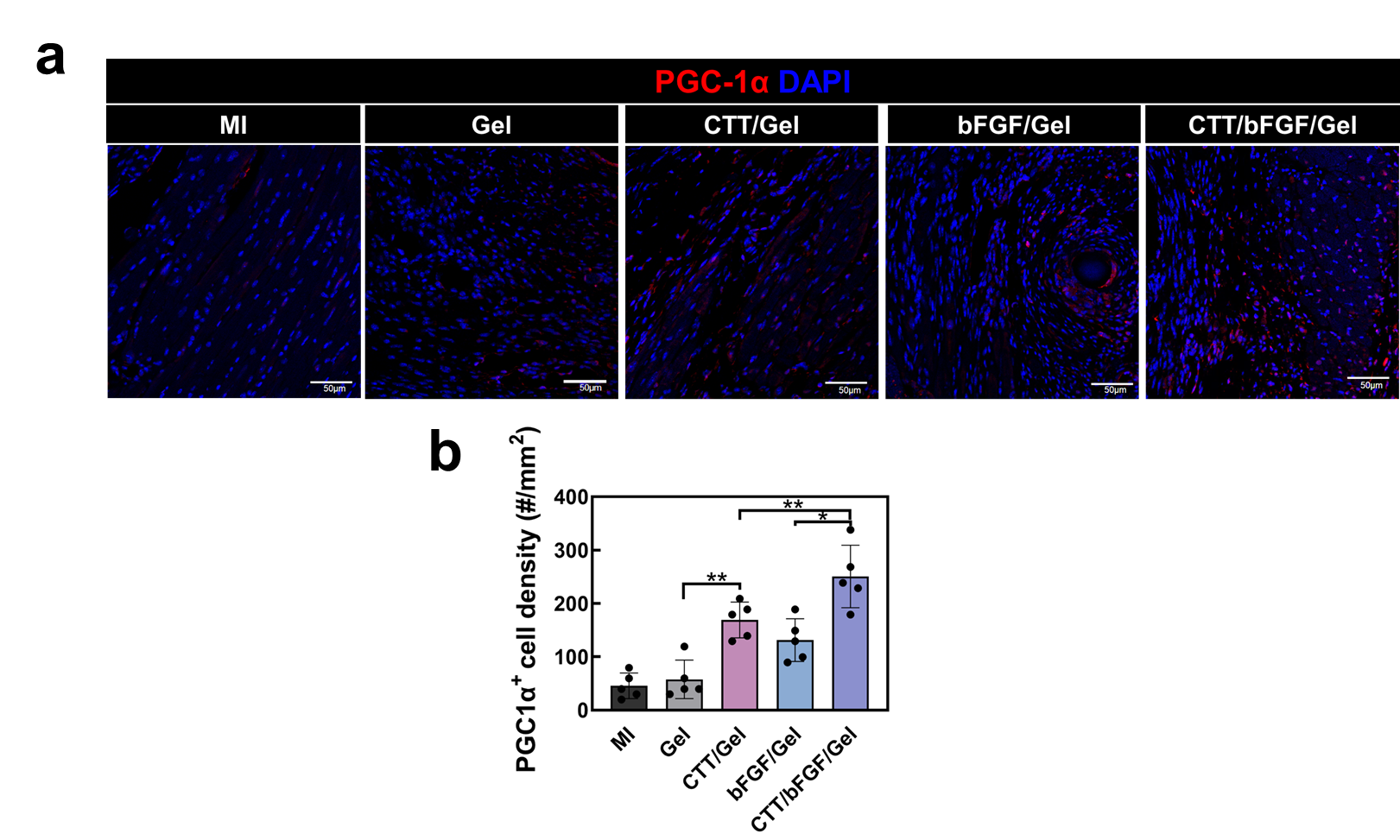


**Figure S3. Effect of CTT, bFGF, and hydrogel on cell metabolism. a.** Representative images for PGC-1α/DAPI staining. **d.** Quantification of PGC-1α^+^ cell density.


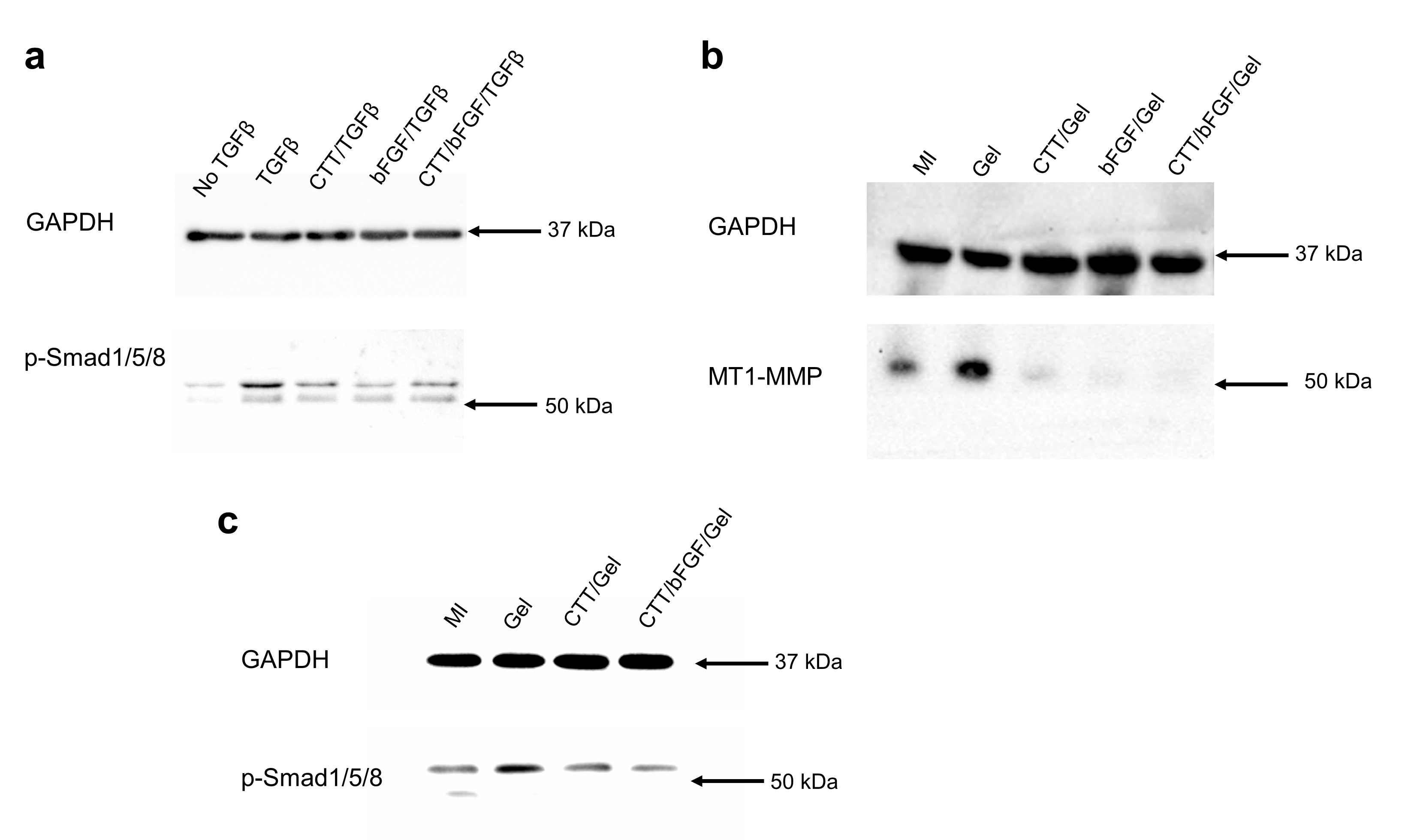


**Figure S4. Uncropped images of immunoblots. a.** Image of blots for Figure 1e. **b.** Image of blots for Figure 4d. **c.** Image of blots for Figure 5f.


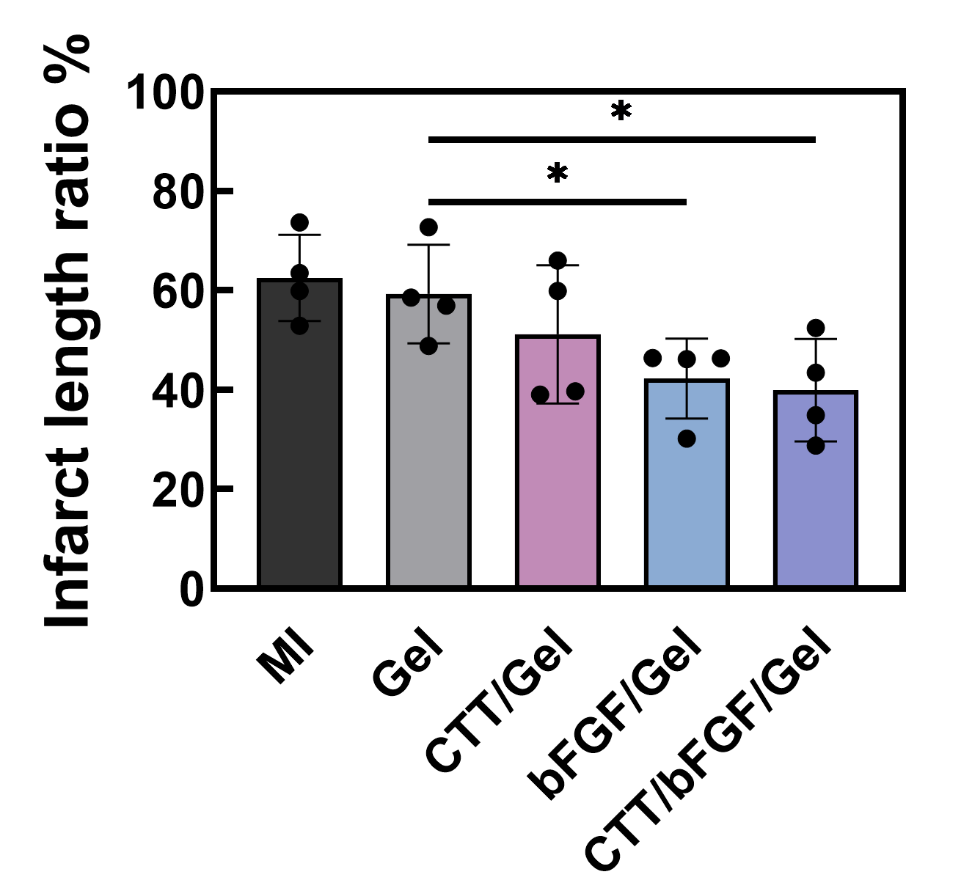


**Figure S5. Quantification of infarct size (infarct length ratio) from H&E images (n=4)**.


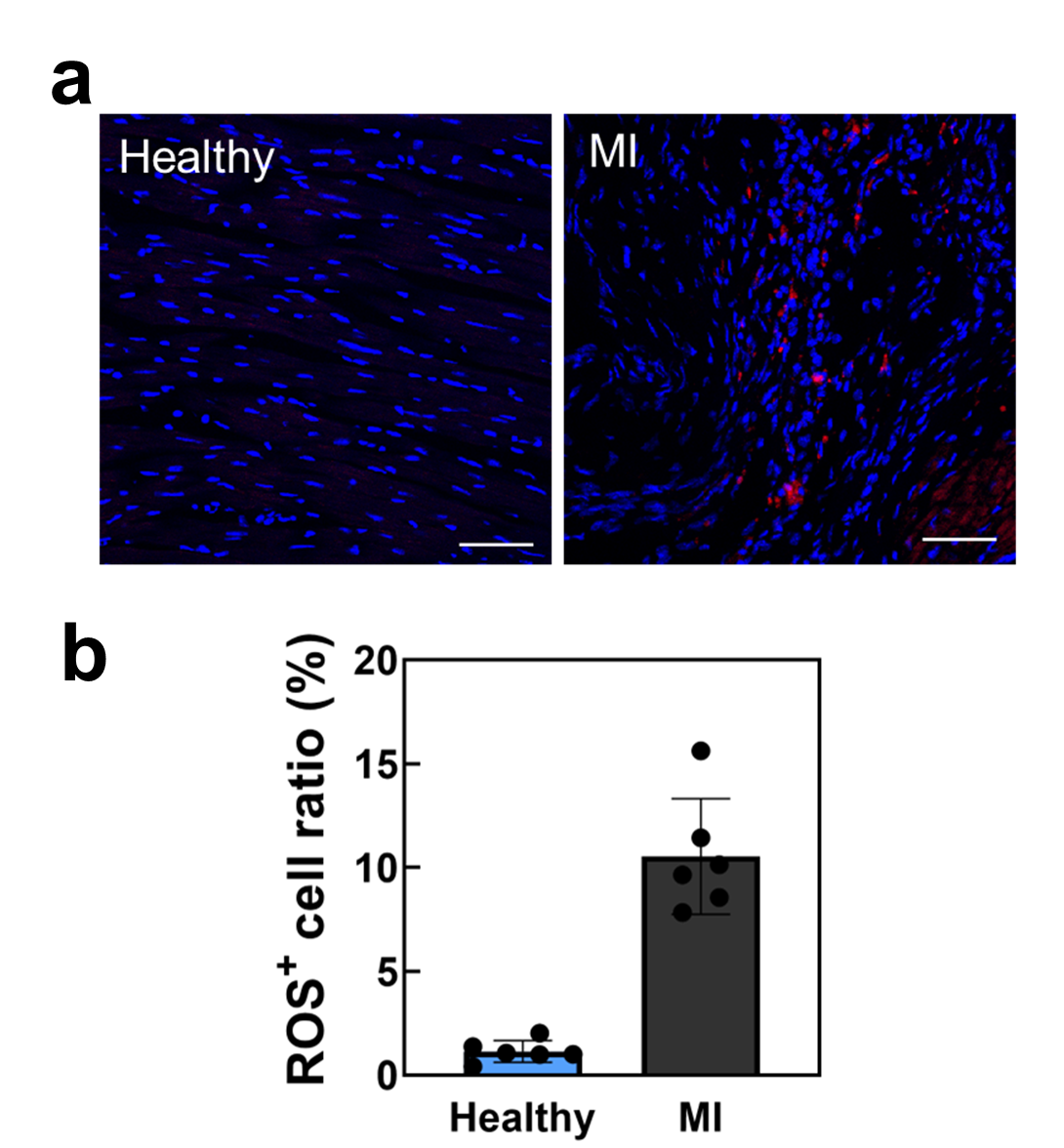


**Figure S6. In vivo ROS detection in ligated hearts at 4 weeks. a.** Representative ROS staining (CM-H2DCFDA) for mice 4 weeks post MI surgery, with healthy mice (no MI surgery) at the same age serving as the control group. Scale bar = 50 µm. **b.** Quantification of the percentage of ROS^+^ cells from the stained images (n=6).


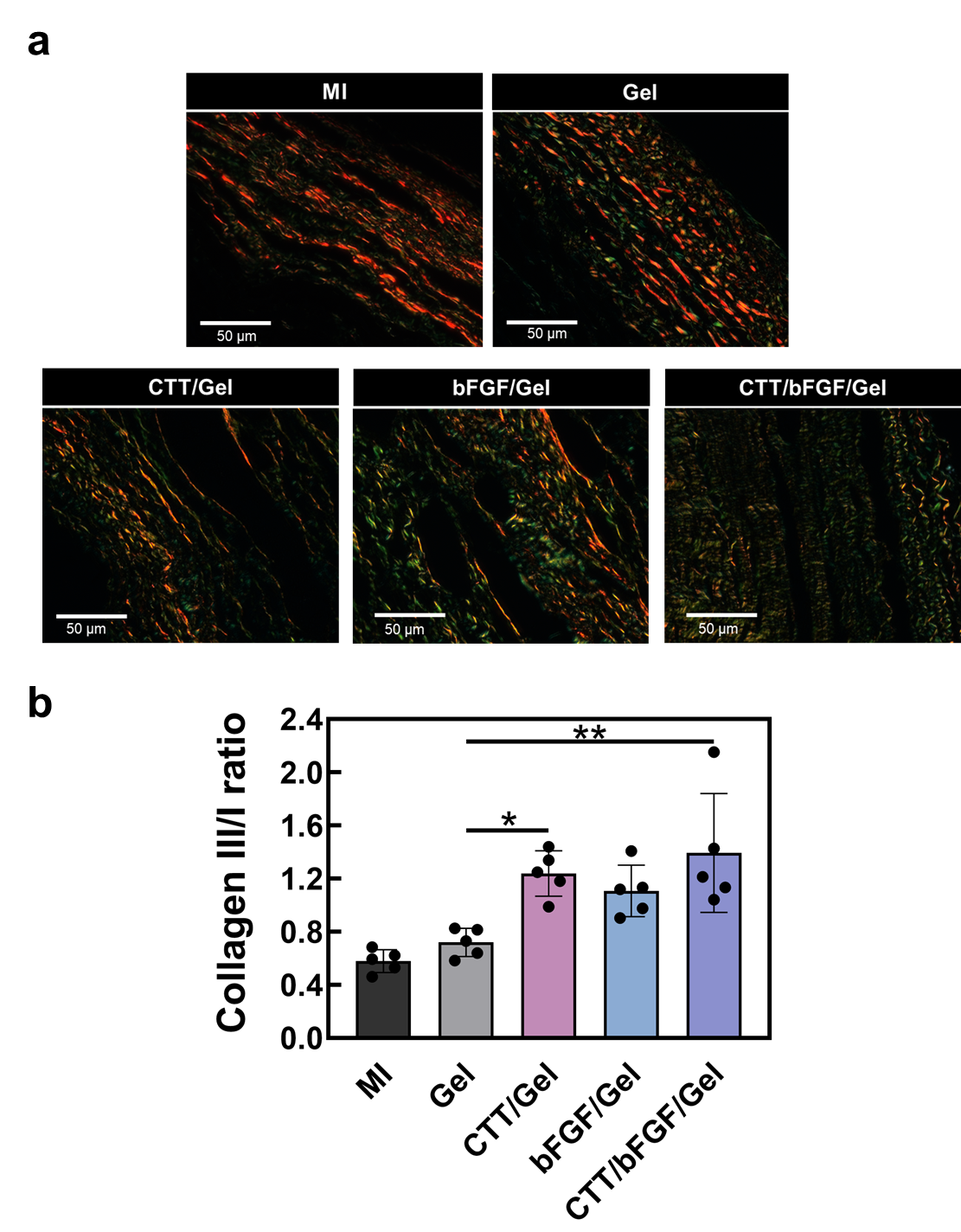


**Figure S7. Polarized PSR image analysis for collagen types I and III.** **a.** Representative images of polarized PSR staining of the infarct heart tissues at day 28. Scale bar=50 μm. **b.** Quantification of the collagen III/I ratio based on the area measurements of the respective regions (n=5).

| **Primer** | **Forward (5’-3’)** | **Reverse (5’-3’)** | **Species** |
| --- | --- | --- | --- |
| *IGF1* | CCCAGAAGGAAGTACATTTG | GTTTAACAGGTAACTCGTGC | Human |
| *VEGFA* | AATGTGAATGCAGACCAAAG | GACTTATACCGGGATTTCTTG | Human |
| *HGF* | CAAGGACCTACGAGAAAATTAC | ATCACAGTTTGGAATTTGGG | Human |
| *PDGFBB* | GGGCAGGGTTATTTAATATGG | AATCAGGCATCGAGACAG | Human |
| *Mmp2* | GAGATCTTCTTCTTCAAGGAC | AATAGACCCAGTACTCATTCC | Mouse |
| *Mmp9* | CTTCCAGTACCAAGACAAAG | ACCTTGTTCACCTCATTTTG | Mouse |
| *Igf1* | CACATCATGTCGTCTTCACACC | GGAAGCAACACTCATCCACAATG | Mouse |
| *Pdgfbb* | GTGGGCAGGGTTATTTAATATG | GAGGGGAACAACATTATCAC | Mouse |

**Table S1.** List of primer sequences used for real-time RT-PCR.
